# Sterile activation of invariant natural killer T cells by ER-stressed antigen-presenting cells

**DOI:** 10.1073/pnas.1910097116

**Published:** 2019-11-05

**Authors:** Melissa Bedard, Dilip Shrestha, David A. Priestman, Yuting Wang, Falk Schneider, Juan D. Matute, Shankar S. Iyer, Uzi Gileadi, Gennaro Prota, Matheswaran Kandasamy, Natacha Veerapen, Gurdyal Besra, Marco Fritzsche, Sebastian Zeissig, Andrej Shevchenko, John C. Christianson, Frances M. Platt, Christian Eggeling, Richard S. Blumberg, Mariolina Salio, Vincenzo Cerundolo

**Affiliations:** ^a^Medical Research Council Human Immunology Unit, Weatherall Institute of Molecular Medicine, University of Oxford, OX3 9DS Oxford, United Kingdom;; ^b^Department of Pharmacology, University of Oxford, OX1 3QT Oxford, United Kingdom;; ^c^Center for Regenerative Therapies, Technische Universität Dresden, 01307 Dresden, Germany;; ^d^Max Planck Institute of Molecular Cell Biology and Genetics, 01307 Dresden, Germany;; ^e^Division of Gastroenterology, Department of Medicine, Brigham and Women’s Hospital Harvard Medical School, Boston, MA 02115;; ^f^Division of Neonatology, Department of Pediatrics, Massachusetts General Hospital, Harvard Medical School, Boston, MA 02114;; ^g^School of Biosciences, University of Birmingham, B15 2TT Egdbaston, United Kingdom;; ^h^Kennedy Institute for Rheumatology, University of Oxford, OX3 7LF Oxford, United Kingdom;; ^i^Department of Medicine I, University Medical Center Dresden, Technische Universität Dresden, 01307 Dresden, Germany;; ^j^Botnar Research Centre, Nuffield Department of Orthopaedics, Rheumatology, and Musculoskeletal Science, University of Oxford, OX3 7LD Oxford, United Kingdom;; ^k^Institute of Applied Optics and Biophysics, 07743 Jena, Germany;; ^l^Department of Biophysical Imaging, Leibniz Institute of Photonic Technologies e.V., 07745 Jena, Germany

**Keywords:** CD1d, ER stress, NKT, cancer

## Abstract

Invariant NKT (iNKT) cells have the unique ability to shape immunity during antitumor immune responses and other forms of sterile and nonsterile inflammation. Recent studies have highlighted a variety of classes of endogenous and pathogen-derived lipid antigens that can trigger iNKT cell activation under sterile and nonsterile conditions. However, the context and mechanisms that drive the presentation of self-lipid antigens in sterile inflammation remain unclear. Here we report that endoplasmic reticulum (ER)-stressed myeloid cells, via signaling events modulated by the protein kinase RNA-like ER kinase (PERK) pathway, increase CD1d-mediated presentation of immunogenic endogenous lipid species, which results in enhanced iNKT cell activation both in vitro and in vivo. In addition, we demonstrate that actin cytoskeletal reorganization during ER stress results in an altered distribution of CD1d on the cell surface, which contributes to enhanced iNKT cell activation. These results define a previously unidentified mechanism that controls iNKT cell activation during sterile inflammation.

## Significance

While there is a clear understanding of how invariant NKT (iNKT) cells are activated in foreign infection, it remains unclear how they are activated during sterile inflammation, including cancer, where they have a well-defined role in tumor immunosurveillance. Here we elucidate a mechanism by which iNKT cells are activated through 1) the presentation of self-lipid antigens by endoplasmic reticulum-stressed antigen-presenting cells and 2) enhanced functional avidity driven by actin cytoskeletal remodeling. We further provide evidence that this mechanism of activation is at play in tumor settings. Here we describe a physiological context, relevant to human health and disease, that drives the presentation of immunogenic self-lipids to activate iNKT cells during sterile inflammation.

Invariant natural killer T (iNKT) cells are a population of innate-like lymphocytes, whose activation is primarily driven by the recognition of lipid antigens presented on monomorphic CD1d molecules (1). In addition to their activation by CD1d-restricted T cell receptor (TCR) engagement, iNKT cells can undergo cytokine-driven activation, mainly mediated by IL-12 and IL-18 (2–4). Activated iNKT cells not only secrete a variety of cytokines, but also enhance CD40L-dependent maturation of dendritic cells (DCs) and activation of B cells (5–7). iNKT cell-dependent DC maturation results in enhanced antigen-specific CD4^+^ and CD8^+^ T cell priming (7, 8), while B cell activation results in enhanced secretion of antibodies and isotype switching (9, 10). Additionally, IFN-γ secretion from iNKT cells drives transactivation of NK cells (11). This influence on multiple immune cell subsets might account for the ability of iNKT cells to exert immunomodulatory roles during infection, autoimmunity, and particularly in antitumor immune responses. Notably, iNKT cell-deficient mice exhibit impaired immunosurveillance and antitumor responses compared to wild-type mice (12). Furthermore, the use of iNKT cell agonists to boost iNKT-mediated antitumor immune responses shows promise as an adjuvant in cancer immunotherapy (7, 13).

Unlike MHC-restricted T cells, iNKT cells also exhibit a degree of basal autoreactivity due to the continuous recognition of endogenous lipids presented by CD1d molecules (14). A more biased Th1 cytokine profile is observed upon Toll-like receptor (TLR)-mediated up-regulation of immunogenic self-lipids (15, 16), direct recognition of bacterial lipids during infection (17), and activation using synthetic glycosidic (18) and nonglycosidic (19) agonists. More recently, we have demonstrated that the tight control by the actin cytoskeleton of the sizes and densities of lipid-loaded CD1d nanoclusters is an additional mechanism modulating iNKT cell activation (20). Importantly, exposure of antigen-presenting cells (APCs) to the TLR 7/8 agonist R848 increases nanocluster density and iNKT cell activation, contributing to the enhanced recognition of CD1d-associated lipids by iNKT cells.

While iNKT cell activation in nonsterile inflammatory conditions is well documented, the mechanisms by which iNKT cell activation is modulated during sterile inflammation, including in cancer, remain unclear. Several pathways might be involved in modulating lipid biosynthesis leading to CD1d-dependent presentation of immunogenic lipid species to drive sterile iNKT cell activation.

We decided to investigate whether the unfolded protein response (UPR) in the endoplasmic reticulum (ER), commonly activated in cancer cells, induces iNKT cell activation. The ER requires a specific subcellular environment in order to execute its main function—to properly fold nascent peptides. Suboptimal conditions in the extracellular milieu, such as hypoxia or nutrient deprivation, can disrupt ER homeostasis and impair its normal function, leading to the accumulation of unfolded or misfolded proteins. This state, termed ER stress, triggers the UPR, which refers collectively to 3 individual yet intersecting pathways that shift the transcriptional and translational profile of the cell in order to restore ER homeostasis (21). The 3 pathways emanate from the 3 sensors in the ER membrane: inositol-requiring enzyme 1 (IRE1), protein kinase RNA-like ER kinase (PERK), and activating transcription factor 6 alpha (ATF6α). Cells undergoing the UPR exhibit marked increase in genes involved in lipid metabolism, including *PPARγ*, *SREB1/2*, and *VLDLR* (22–24). In hepatic diseases—such as nonalcoholic fatty liver disease, nonalcoholic steatohepatitis, and hepatic steatosis—ER stress contributes greatly to dysregulated lipid metabolism (23, 24). Notably, iNKT cells contribute to the sterile inflammatory component of these pathologies (25, 26). ER stress also is a hallmark in a variety of cancers, including multiple myeloma, which can be treated with bortezomib, a proteasome inhibitor that further enhances ER stress in the malignant cells (27).

In this paper, we demonstrate that ER-stressed APCs lead to CD1d-dependent iNKT cell activation. We identify the PERK pathway as the main regulator of this response and demonstrate that lipid fractions isolated from ER-stressed wild-type, but not from PERK knockdown (KD) cells, reconstitute iNKT cell activation in plate-bound assays. Furthermore, we demonstrate that ER stress modulates actin cytoskeletal reorganization, resulting in an altered distribution of CD1d on the cell surface, contributing to enhanced iNKT cell activation. These results demonstrate a mechanism of iNKT cell activation in sterile inflammatory conditions.

## Results

### ER-Stressed APCs Activate iNKT Cells in a CD1d- and UPR-Dependent Manner.

**Fig. 1.**
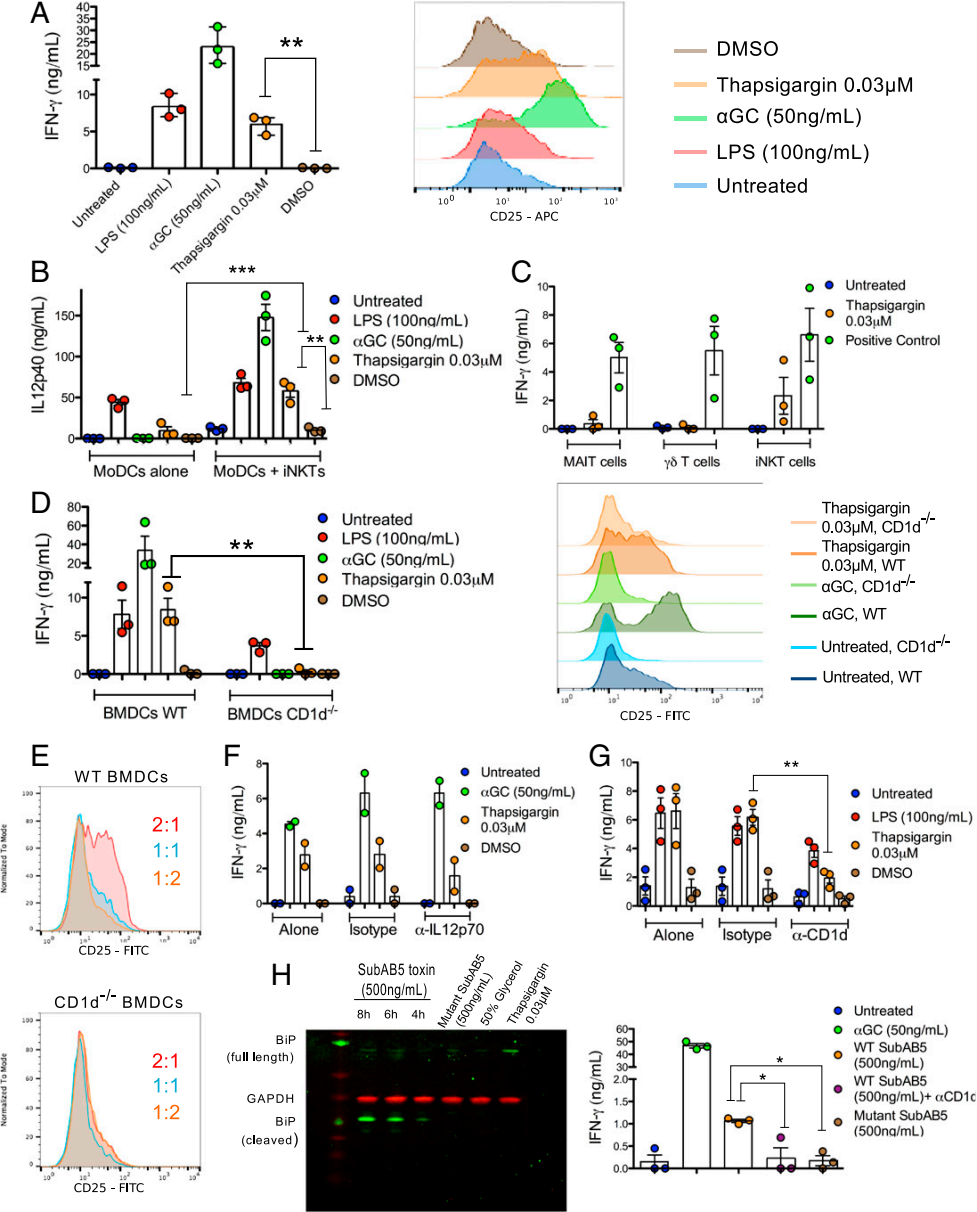
ER-stressed APCs activate iNKTs cells in a CD1d- and UPR-dependent manner. (*A*) Human MoDCs treated as indicated were cocultured with human iNKT cells. iNKT cell activation was measured by secretion of IFN-γ from iNKT cells (*Left*) and by increased surface expression of CD25 on iNKT cells (*Right*). **P < 0.005 by an unpaired, 2-tailed *t* test. IFN-γ secretion is the average of *n* = 3 biological replicates, and the CD25 histograms are representative of *n* = 3 biological replicates. (*B*) MoDCs treated as indicated were either left untreated (MoDCs alone) or cocultured with human iNKT cells. MoDCs maturation is measured by IL12p40 secretion in the supernatants. **P < 0.005 and ***P < 0.001 by a 1-way ANOVA with a Bonferroni posttest. IL12p40 secretion is the average of *n* = 3 biological replicates. (*C*) MoDCs were either left untreated, treated with thapsigargin, α-galactosylceramide (αGC) (50 ng/mL), 5ARU (1 μg/mL) + Methyl Glyoxal (50 μM), or HMBPP (1 μM) as positive controls and cocultured with either iNKT, MAIT, or γδ T cells. IFN-γ in the supernatants of these cocultures was measured by ELISA. IFN-γ secretion is the average of *n* = 3 biological replicates. (*D*) BMDCs from wild-type and CD1d^−/−^ mice were cocultured with human iNKT cells. iNKT cell activation was measured by IFN-γ secretion (*Left*) and CD25 up-regulation (*Right*). **P < 0.005 by an unpaired, 2-tailed *t* test. IFN-γ secretion is the average of *n* = 3 biological replicates, and the CD25 histograms are representative of *n* = 3 biological replicates. (*E*) Human iNKT cells were cocultured with a decreasing proportion of thapsigargin-treated wild-type or CD1d^−/−^ BMDCs (BMDCs:iNKTs) and iNKT cell activation was assessed by CD25 up-regulation. These histograms are representative of *n* = 3 biological replicates. (*F*) MoDCs treated as indicated were cocultured with human iNKT cells in the presence of IL12p70 blocking antibody (20 μg/mL) or the appropriate isotype control. IFN-γ secretion is the average of *n* = 2 biological replicates. (*G*) THP1 cells treated as indicated then cocultured with human iNKT cells in the presence of CD1d blocking antibody (20 μg/mL) or the appropriate isotype control. **P < 0.005 by an unpaired, 2-tailed *t* test. IFN-γ secretion is the average of *n* = 3 biological replicates. (*H*) ER stress in THP1 cells treated with the SubAB5 toxin at the indicated concentration for different timepoints is demonstrated by cleavage of the full-length BiP protein, and detection of the BiP cleavage product by Western blot. The inactive mutant SubAB5 toxin was used as a negative control. Activation of human iNKT cells cocultured with SubAB5-pretreated THP1 wild type cells in the presence of absence of CD1d blocking antibody (20 μg/mL) was measured by IFN-γ secretion (*Right*). In both panels, the inactive mutant SubAB5 toxin was used as a negative control. *P < 0.05 by an unpaired, 2-tailed *t* test. IFN-γ secretion is the average of *n* = 3 biological replicates.

To address whether ER-stressed CD1d^+^ APCs could activate iNKT cells in the absence of either synthetic iNKT cell agonists, TLR agonists, or pathogens, human monocyte-derived DCs (MoDCs) were treated with the ER stress-inducing agent, thapsigargin, which blocks the sarco-ER calcium pump (28). After thapsigargin treatment at the optimized dose of 0.03 μM (*SI Appendix*, Fig. S1*A*), MoDCs were washed to remove residual drug and cocultured with human iNKT cells overnight. We observed that iNKT cells cocultured with the ER-stressed MoDCs secreted IFN-γ (Fig. 1 *A*, *Left*) and up-regulated expression of CD25 (Fig. 1 *A*, *Right*), indicating enhanced iNKT cell activation, compared to iNKT cells cocultured with untreated or DMSO-treated MoDCs. Furthermore, thapsigargin-treated MoDCs cocultured with iNKT cells secreted more IL12p40 than thapsigargin-treated MoDCs alone (Fig. 1*B*), consistent with their iNKT cell-dependent enhanced maturation. The concentration of thapsigargin used in this and following experiments stemmed from a titration curve, where the lowest dose tested, 0.03 μM, elicited the greatest iNKT cell response, likely as result of improved cell survival (*SI Appendix*, Fig. S1*A*). At this concentration, iNKT cells also secreted IL-4, GM-CSF, and IL-13 (*SI Appendix*, Fig. S1*B*). Importantly, the effect of thapsigargin seemed to be specific to CD1d-restricted lymphocytes, as defined by the lack of activation of mucosal associated invariant T cells and Vγ9Vδ2 T cells (Fig. 1*C*).

To confirm that thapsigargin treatments induced ER stress and triggered the UPR, we treated the CD1d^+^ monomyelocytic cell line THP1 with a similar range of concentrations used to treat MoDCs and analyzed THP1 cells for increased expression of UPR markers by Western blot. At a concentration of 0.03 μM in THP1 cells, thapsigargin up-regulated the chaperones binding immunoglobulin protein (BiP) and protein disulfide isomerase (PDI), as well as the UPR transcription factor C/EBP homologous protein (CHOP), which lies downstream of the PERK branch (*SI Appendix*, Fig. S1*C*). Furthermore, the low concentration of thapsigargin was sufficient to induce splicing of XBP1 mRNA (*SI Appendix*, Fig. S1*D*), reflecting the excision of a 26-base pair intron by the IRE1 endoribonuclease to produce the active XBP1 transcription factor responsible for restoring ER homeostasis. Taken together, these results indicate that the 0.03-μM concentration of thapsigargin is sufficient to induce ER stress that activates the UPR.

We wanted to know whether this phenotype was restricted to human myeloid cells or could be extended to other cell types and to murine cells. We were particularly interested in whether tumor cells, which typically are prone to ER stress, could activate iNKT cells upon UPR activation. To this end, we demonstrated that that the murine Lewis Lung carcinoma (3LL line, which expresses endogenously CD1d molecules) activated iNKT cells upon treatment with thapsigargin, which was further enhanced upon transduction with lentiviral vectors encoding CD1d molecules (*SI Appendix*, Fig. S1*E*).

To test whether activation of iNKT cells upon coculture with thapsigargin-treated APCs is CD1d-dependent, bone marrow (BM) cells were harvested from either wild-type or CD1d^−/−^ C57BL/6 mice and differentiated into BM-derived DCs (BMDCs), which were then treated with thapsigargin, as previously described, washed, and cocultured with human iNKT cells. While thapsigargin-treated wild-type BMDCs induced IFN-γ secretion and CD25 up-regulation on iNKT cells, thapsigargin-treated CD1d^−/−^ BMDCs failed to activate iNKT cells (Fig. 1*D*) at any ratio tested (Fig. 1*E*).

To determine the contributions of TCR-dependent activation (signal 1) and cytokine-driven activation (signal 2) in driving ER stress APC-mediated iNKT cell activation, we tested whether blockade of IL12p70 or CD1d would reduce iNKT cell activation to ER-stressed MoDCs. While iNKT cell activation was only partially reduced with the addition of an IL12p70 blocking antibody (Fig. 1*F*), addition of a CD1d-blocking antibody to ER-stressed THP1 cells almost abolished iNKT cell activation (Fig. 1*G*). In addition, we ruled out the possibility that CD1d up-regulation accounted for iNKT cell activation during ER stress, as surface expression of CD1d molecules did not increase on thapsigargin-treated wild-type or CD1d-overexpressing THP1 cells (*SI Appendix*, Fig. S1*F*). Similarly, surface expression of ICAM1, which is reported to activate iNKT cells through binding to LFA-1 (29), did not significantly increase on the cell surface of thapsigargin-treated MoDCs when measured by flow cytometry (*SI Appendix*, Fig. S1*G*). However, by stimulated emission depletion (STED) microscopy, we observed an increase in ICAM1 mean intensity per unit area, which might suggest that ICAM1 is also rearranged in thapsigargin-treated THP1-CD1d cells (*SI Appendix*, Fig. S1*H*).

Since thapsigargin alters the distribution of calcium ions within the cell, it has the potential to cause off-target effects unrelated to the UPR. To provide further support for a link between CD1d-dependent iNKT cell activation and the UPR, we treated THP1 cells with the subtilase cytotoxin (SubAB5 toxin), which specifically cleaves the sentinel UPR protein, BiP, resulting in a 28-kDa cleaved BiP fragment (30). Cleaved BiP is unable to bind to the luminal domain of each of the 3 sensors of the UPR, and thus mimics conditions that trigger the UPR. We confirmed that the SubAB5 toxin induced the UPR, as defined by cleavage of BiP in THP1 cells (Fig. 1 *H*, *Left*) and that SubAB5 toxin-treated THP1 cells were able to induce iNKT cell activation in a CD1d-dependent manner (Fig. 1 *H*, *Right*). Importantly, as a negative control we used an inactive mutant of the SubAB5 toxin, with a single amino acid mutation in the toxin enzymatic cleft (31) that prevents BiP cleavage (Fig. 1 *H*, *Left*), and demonstrated its inability to induce iNKT cell activation (Fig. 1 *H*, *Right*). Altogether, these results demonstrated that ER-stressed human and mouse APCs mediate iNKT cell activation in a CD1d-dependent and UPR-dependent manner.

### Activation of iNKT Cells by ER-Stressed APCs Requires Signaling through the PERK Pathway to Direct CD1d-Presentation of Immunogenic Self-Lipid Antigens.

**Fig. 2.**
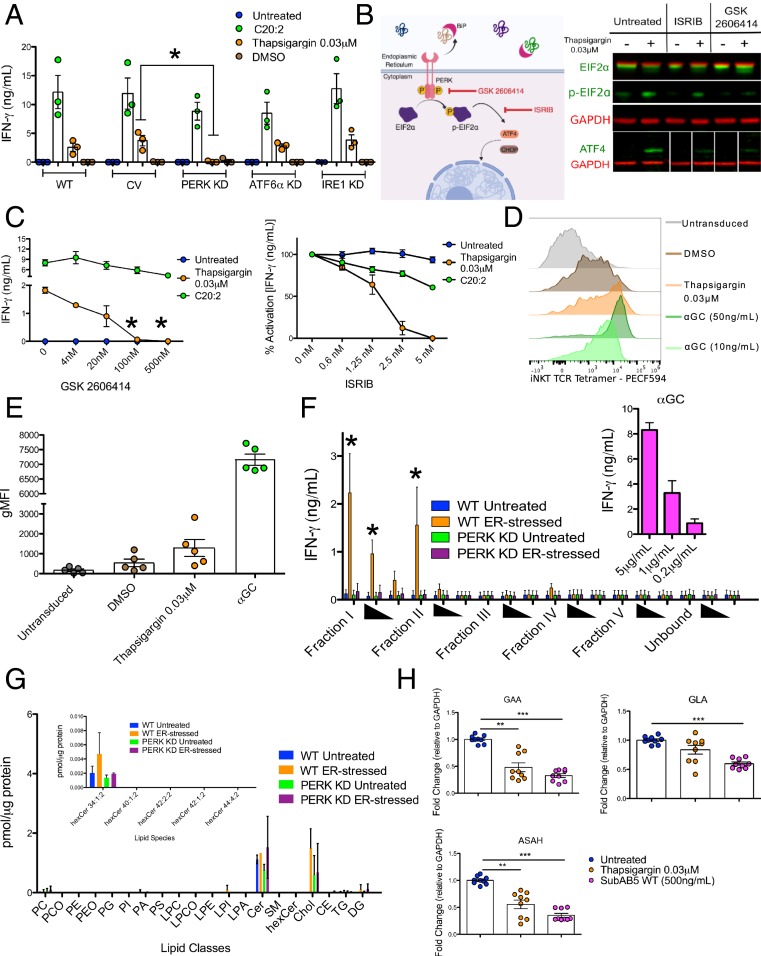
Activation of iNKT cells by ER-stressed APCs requires signaling through the PERK pathway to direct CD1d-presentation of immunogenic self-lipid antigens. (*A*) Wild-type, control vector (CV) and IRE1, ATF6α, or PERK KD vector transduced THP1 wild-type cells were cocultured with human iNKT cells and activation was assessed by IFN-γ secretion. *P < 0.05 by a 1-way ANOVA with a Dunnett’s multiple comparison posttest. IFN-γ secretion is the average of *n* = 3 biological replicates. (*B*) An illustration of the mechanism of action of 2 inhibitors of the PERK pathway: The PERK kinase inhibitor GSK 2606414 and the phospho-eIF2α inhibitor ISRIB. Validation of ISRIB and GSK2606414 in blocking PERK signaling via Western blot of THP1 cells after 6 h of treatment. ISRIB blocks PERK signaling events beyond eIF2α phosphorylation (i.e., ATF4 up-regulation). GSK2606414 blocks eIF2α phosphorylation and ATF4 up-regulation (*Right*). (*C*) Effect of GSK2606144 (*Left*) and ISRIB (*Right*) on iNKT cell activation by thapsigargin-treated THP1 cells. THP1 cells pulsed with C20:2, an analog of αGC that binds surface CD1d molecules were used a control for nonspecific inhibitor toxicity and functional CD1d presentation. *P < 0.05 by a 1-way ANOVA with a Dunnett’s multiple comparison posttest. The starred data points are compared to the thapsigargin condition without inhibitor added. The data points represent the average of *n* = 3 biological replicates. (*D*) Representative FACS plots of untransduced THP1 wild-type cells (gray) or thapsigargin-treated THP1-CD1d overexpressing cell stained using a fluorescently labeled, soluble iNKT-TCR tetramer. As a control, THP1-CD1d overexpressing cells were treated with 2 concentrations of αGC. (*E*) Cumulative data. Each dot of depicts the geometric mean fluorescence intensity of the TCR staining of 1 of *n* = 5 biological replicates, each performed in technical duplicates. (*F*) Lipids extracted from untreated or thapsigargin-treated wild-type or PERK KD THP1 cells as described in the methods were pulsed in 3 decreasing concentrations (solid triangles) onto recombinant, plate-bound CD1d molecules and cocultured with human iNKT cells. Activation is measured by IFN-γ secretion. As a control αGC was added to the CD1d-coated plates (*Inset*). *P < 0.05 by 1-way ANOVA with a Bonferroni posttest comparing ER-stressed wild-type group with the untreated wild-type group IFN-γ secretion is the average of *n* = 5 biological replicates. (*G*) The lipid classes and species in fraction I were identified and quantified by shotgun MS. Lipid quantity (in picomoles) was normalized to protein concentration for each sample. (*H*) Fold-change in transcriptional expression of the enzymes GAA, GLA, and ASAH1 relative to GAPDH in unstressed and ER-stressed MoDCs. Each dot represents 1 of 3 technical replicates for an *n* = 3 biological replicates. **P < 0.005 and ***P < 0.001 by a Mann–Whitney *U* test.

Having demonstrated that ER-stressed APC-mediated iNKT cell activation is UPR-dependent, we next determined which of the 3 UPR pathways—IRE1, PERK, and ATF6α—contributes to this phenotype. We lentivirally transduced wild-type THP1 cells with short-hairpin RNAs (shRNAs) targeting each of the sentinel proteins of the 3 pathways: IRE1, PERK, and ATF6α. We first confirmed a reduced expression of each of the 3 sensors via Western blot (*SI Appendix*, Fig. S2*A*) and then demonstrated in functional assays that only PERK KD THP1 cells failed to induce iNKT cell activation upon thapsigargin treatment, when compared to untransduced and control vector-transduced THP1 cells (Fig. 2*A*). As a control, we showed that CD1d expression on the surface PERK KD cells, both in wild-type and CD1d-overexpressing THP1 cells, was not reduced (*SI Appendix*, Fig. S2*B*). These findings indicate that the PERK pathway contributes to ER-stressed APC-mediated iNKT cell activation without altering levels of CD1d surface expression.

To further interrogate the role of the PERK pathway, we cotreated THP1 cells with thapsigargin and small-molecule inhibitors that block the PERK signaling cascade at different points: 1) GSK2606414, which inhibits the PERK autophosphorylation step that follows PERK dimerization upon BiP unbinding; and 2) integrated stress response inhibitor (ISRIB), which blocks signaling from phospho-elongation factor 2α (eIF2α) and therefore blocks the downstream selective translational inhibition characteristic of the PERK pathway (schematically depicted in Fig. 2*B*). We observed a dose-dependent decrease in iNKT cell activation mediated by thapsigargin-treated THP1 cells in the presence of both GSK2606414 (Fig. 2 *C*, *Left*) and ISRIB (Fig. 2 *C*, *Right*). Small-molecule inhibitors that block IRE1 function, and therefore prevent XBP1 splicing and downstream transcription, had no effect on ER stress-mediated iNKT cell activation (*SI Appendix*, Fig. S2*E*). Similarly, Ceapin-A7, a small-molecule inhibitor that blocks ATF6α signaling, had no effect on ER stress-mediated iNKT cell activation (*SI Appendix*, Fig. S2*F*).

Finally, we ruled out a role for the well-characterized transcription factor ATF4, which is translated upon eIF2α signaling, as ATF4 KD and control vector-transduced THP1 cells treated with thapsigargin did not differ in their capacity to activate iNKT cells (*SI Appendix*, Fig. S2*C*). Since ER stress, particularly through components of the PERK pathway, can trigger autophagy (31), we cocultured human iNKT cells with thapsigargin-treated BMDCs from wild-type or Vav-Cre ATG7^fl/fl^ mice. ER stress-mediated iNKT cell activation was comparable in BMDCs derived from both wild-type and Vav-Cre ATG7^fl/fl^ mice, suggesting that autophagy does not contribute to the phenotype (*SI Appendix*, Fig. S2*D*).

We next reasoned that iNKT cell activation upon coculture with ER-stressed APCs might be accounted for by change in the repertoire of lipids presented by CD1d-molecules under ER stress. To test this hypothesis, we probed the surface of THP1 cells overexpressing CD1d molecules (THP1-CD1d cells) with a fluorescently labeled iNKT-TCR tetramer, which detects antigenic self-lipids presented by CD1d molecules (16). THP1-CD1d cells treated with thapsigargin exhibited increased tetramer staining compared to controls (Fig. 2 *D* and *E*). Together these findings indicate that ER stress modulates immunogenic self-lipid presentation by CD1d molecules.

To gain further insights into the identity of immunogenic self-lipid antigens presented by CD1d molecules by ER-stressed THP1 cells, we extracted lipids from thapsigargin-treated cells and separated them using amino-propyl columns into 6 different fractions based on hydrophobicity. Each lipid fraction was then pulsed onto recombinant CD1d monomers in a CD1d plate-bound assay, allowing us to evaluate the direct contribution of PERK-regulated lipids bound to CD1d in driving iNKT cell activation, while removing confounding variables including APC cytokine secretion and cell death. Of the 6 fractions, lipids in fractions I and II from ER-stressed wild-type THP1 cells activated iNKT cells in a dose-dependent manner (Fig. 2*E*). In contrast, lipids from the same fractions of ER-stressed PERK KD THP1 cells failed to activate iNKT cells (Fig. 2*E*). Furthermore, we confirmed that such activating lipids were presented by CD1d molecules by using a CD1d blocking antibody in the assay, which reduced iNKT cell activation (*SI Appendix*, Fig. S2*G*). Mass spectrometry (MS) analysis confirmed the lipid classes in the activating fraction and their relative abundance (Fig. 2*F*).

Endogenous glycosphingolipids (GSLs) are among the classes of lipids eliciting increased iNKT cell autoreactivity (32). To prevent autoimmunity, degradation and synthesis of endogenous GSLs is tightly regulated. For example, in TLR-stimulated DCs a transient down-regulation of the activity of lysosomal enzymes that catabolize GSLs occurs, resulting in increased iNKT cell activation (33). We therefore sought to determine if induction of ER stress modulates expression of these enzymes. We observed that treatment of MoDCs with either thapsigargin or the SubAB5 toxin reduced transcription of 3 main GSL-degrading enzymes: α-glucosidase (GAA), α-galactosidase (GLA), and acid ceramidase (ASAH1) (Fig. 2*G*), potentially leading to accumulation of stimulating GSLs.

Together, these results demonstrate that enhanced iNKT cell activation upon coculture with ER-stressed APCs is mediated by the PERK-dependent presentation of antigenic self-lipids from fractions I and II by CD1d molecules.

### The Immunogenic Self-Lipid Antigens Are Loaded onto CD1d Molecules in the Endosomal/Lysosomal Recycling Pathway in ER-Stressed APCs.

**Fig. 3.**
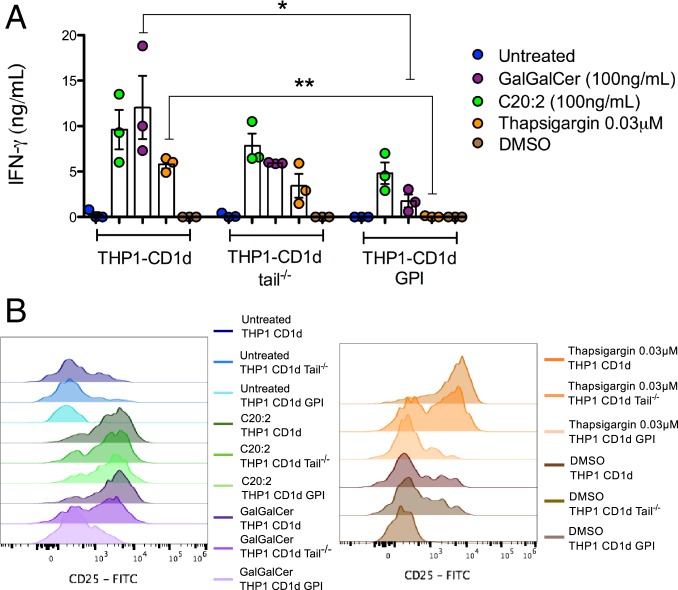
The immunogenic self-lipid antigens are loaded onto CD1d molecules in the endosomal/lysosomal recycling pathway in ER-stressed APCs. Thapsigargin-treated THP1 cells overexpressing full length CD1d (THP1-CD1d), CD1d with a deletion of 11 amino acids in the cytoplasmic tail domain (THP1-CD1d tail^−/−^) and GPI-linked CD1d (THP1-CD1d GPI) were cocultured with human iNKT cells. As a control, the THP1 cell lines were treated with C20:2. iNKT cell activation was measured by (*A*) IFN-γ secretion and (*B*) CD25 surface expression. Experiments are representative of *n* = 3. *P < 0.05 and **P < 0.005 by a 1-way ANOVA with a Dunnett’s multiple comparison posttest.

We aimed to determine in which subcellular compartment such endogenous ligands presented by ER-stressed APCs are loaded onto CD1d molecules. For this purpose, we used 3 THP1 cell lines that overexpressed different variants of CD1d molecules: 1) The THP1 cell line overexpressed full-length CD1d, which can traffic normally to the cell surface and through the endosomal/lysosomal recycling compartments; 2) the THP1 cell line overexpressed CD1d molecules lacking 10 amino acids in the cytosolic tail (THP1 CD1d-tail^−/−^) (34), which can traffic to the surface and early endosome, but not to the lysosomal compartment; 3) the THP1 cell line overexpressed CD1d molecules with glycosylphosphatidylinositol (GPI) anchors (THP1-CD1d GPI) that are retained at the cell surface upon ER-loading and are unable to enter the recycling pathway. In comparison to ER stress-mediated iNKT cell activation elicited by THP1-CD1d cells, we observed a partial reduction in thapsigargin-mediated iNKT cell activation upon coculture with THP1 CD1d-tail^−/−^, and complete abrogation of thapsigargin-mediated iNKT cell activation upon coculture with THP1-CD1d GPI cells (Fig. 3). To control for equal levels of functional surface CD1d, we demonstrated that the iNKT cell agonist C20:2 (35), which can be directly presented by surface CD1d molecules, was efficiently presented by all 3 THP1 cell lines. As an additional control, we included Gal(α1-2)galactosylceramide (GalGalCer) (36), which requires processing and loading in the acidic lysosome to be recognized by iNKT cells. While THP1-CD1d cells were able to present GalGalCer and activate iNKT cells, THP1-CD1d tail^−/−^ and THP1-CD1d GPI cells pulsed with GalGalCer either reduced activation of iNKT cells or failed to activate them, respectively, similar to the thapsigargin condition. This finding indicates that the activating self-lipid antigens in ER-stressed APCs are loaded during CD1d recycling in the endosome and lysosome.

### Actin-Mediated CD1d Reorganization Contributes to iNKT Cell Activation by ER-Stressed APCs.

**Fig. 4.**
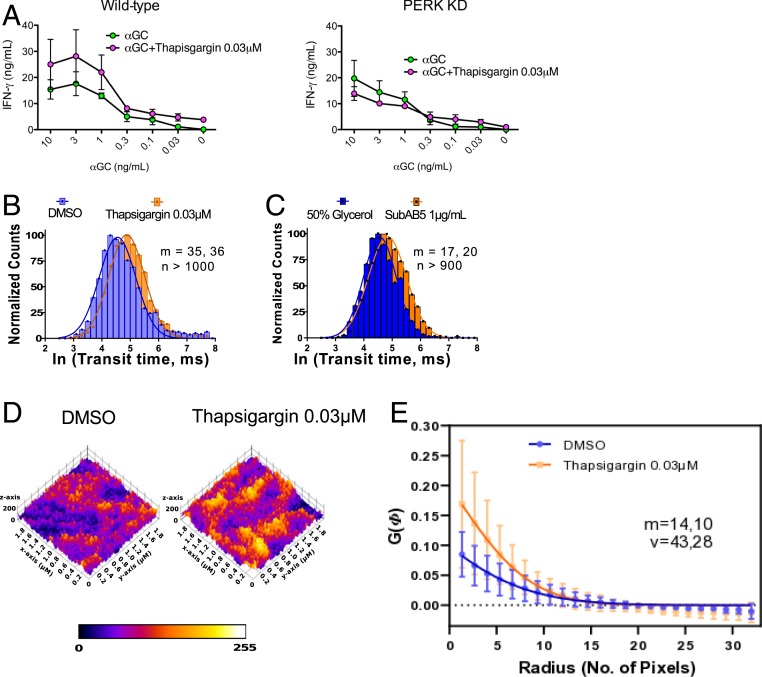
ER stress changes plasma membrane dynamics leading to heterogeneous distribution of CD1d in the plasma membrane of THP1 CD1d cells. (*A*) iNKT cells activation, measured by IFN-γ secretion, induced by wild-type (*Left*) and PERK KD (*Right*) THP1 cells pretreated with thapsigargin 0.03 μM and/or pulsed 3 h with a dose curve of αGC. Experiments are representative of *n* = 2. (*B* and *C*) show the normalized frequency distribution of ln (transit time, ms) of CD1d measured from the basal plane of the cells and analyzed as described in the sFCS analysis section. At least 3 independent measurements were taken to report the values obtained from sFCS data. (*D*) The color-coded topological distribution of CD1d for a 2 × 2-μm region is shown using fire LUT of Fiji for the different treatment conditions. (*E*) Quantification of heterogeneity in CD1d distribution on the surface of DMSO and thapsigargin-treated THP1-CD1d cells using the spatial autocorrelation function. m, no. of cells; n, no. of curves; v, no. of autocorrelation curves.

Given the established link between formation of large CD1d nanocluster and iNKT cell activation to self-lipid antigens through increased TCR-CD1d avidity (20), we investigated whether ER stress not only contributes to the presentation of immunogenic self-lipid antigens on CD1d, but also by increasing functional avidity of iNKT-TCR binding to CD1d molecules loaded with agonist lipid antigens. We tested whether thapsigargin pretreated THP1 wild-type cells pulsed with decreasing doses of αGC would exhibit enhanced iNKT cell activation. We found that pretreatment with thapsigargin did increase iNKT cell activation to αGC across a range of concentrations (Fig. 4*A*). In addition, no such enhancement in iNKT cell activation was observed when PERK KD THP1 cells were used as APC, while they comparably stimulated iNKT cells upon αGC pulsing (Fig. 4*A*).

This observation suggests that ER stress can enhance iNKT cell activation by an additional lipid antigen-independent mechanism and led us to further investigate changes in CD1d distribution and functional avidity by quantifying their diffusion and interaction dynamics using scanning fluorescence correlation spectroscopy (sFCS) (37, 38). sFCS measurements allow for highly accurate determination of the transit time of CD1d molecules through a given observation spot to generate average diffusion coefficients. For this, we stained THP1-CD1d cells with fluorescently labeled CD1d Fab antibodies and performed sFCS measurements at the basal plane of the cells away from the periphery using confocal microscopy. Consistently, for both thapsigargin-treated APCs (Fig. 4*B*) and SubAB5 toxin-treated THP1 cells (Fig. 4*C*), we found that CD1d molecules exhibited a global decrease in their average transit time and thus slower free diffusion and reduced mobility compared to the untreated control conditions (*SI Appendix*, Fig. S3*A*). Since a global decrease in mobility could reflect a more structured organization of CD1d and increased functional avidity, we analyzed the CD1d distribution on the surface of fixed ER-stressed cells via STED imaging, using fluorescently labeled CD1d-specific Fab antibodies. This analysis revealed an altered landscape of CD1d on the surface of ER-stressed THP1-CD1d cells compared to the DMSO control, consistent with the possibility that ER stress induces a heterogeneous distribution of CD1d on the cell surface (Fig. 4*D*). To further quantify the spatial reorganization, we applied a spatial autocorrelation function to the STED fluorescence data, a method successfully used to characterize heterogeneity and oligomerization in protein organization on immune cell surfaces (39).

There was no statistical difference in the amplitude of the spatial autocorrelation function for thapsigargin-treated THP1-CD1d cells compared to the DMSO control (Fig. 4*E*); however, a trend indicated a more heterogeneous fluorescent signal and thus a tendency toward a more confined spatial CD1d distribution on the cell surface. Similarly, THP1-CD1d GPI cells exhibited a similar pattern of spatial CD1d distribution (*SI Appendix*, Fig. S3*B*), suggesting that this was likely driven by changes in the actin cytoskeleton upon ER stress (40–42). However, due to the lack of activating self-lipid antigens stemming from impaired endosomal/lysosomal recycling, these cells do not induce appreciable iNKT cell activation. In agreement with enhanced staining at the cell surface with iNKT TCR tetramers, we also observed enhanced staining by confocal microscopy with iNKT-TCR monomers, which might reflect enhanced avidity as well as increased affinity for an immunogenic self-lipid in ER-stressed cells (*SI Appendix*, Fig. S3*C*).

**Fig. 5.**
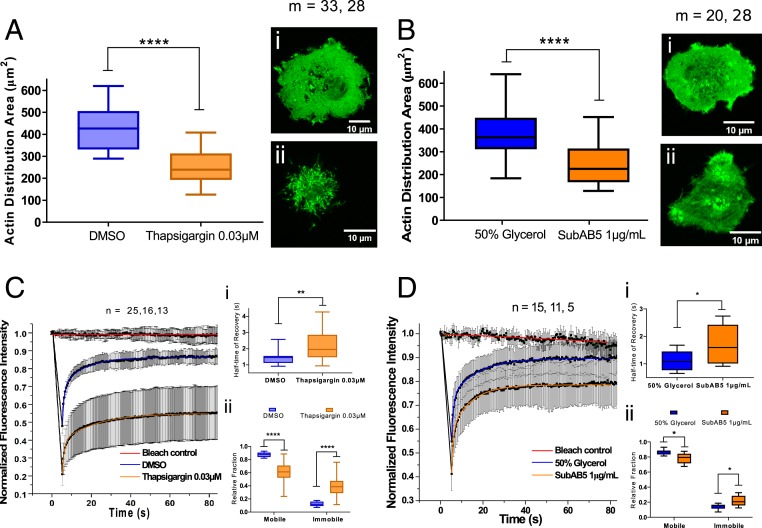
ER stress alters cell morphology, actin turnover dynamics, and intracellular actin filament organization in THP1 CD1d cells. Cells were treated with appropriate control solvent (DMSO or Glycerol) or ER stress-inducing drugs (thapsigargin 0.03 μM or 1 μg/mL SubAB5). (*A* and *B*) microscopic images of actin fluorescence for quantification of actin distribution area. (*i*) Image of a cell treated with the solvent control; (*ii*) the corresponding ER-stress condition. (*C* and *D*) FRAP analysis of fluorescence recover of control or ER-stressed THP1 cells to measure actin filament polymerization dynamics. Panel (*i*) shows half-time of fluorescence recovery and (*ii*) compares the mobile an immobile actin fractions upon treatment. The data represent *n* ≥ 3 biological replicates. *P < 0.05, **P < 0.005, ****P < 0.0001 by unpaired, 2-tailed *t* test.

Next, we sought to further dissect the processes leading to the spatial reorganization of CD1d. We previously demonstrated that the spatial organization and dynamics of the cortical actin cytoskeleton beneath the membrane influences the diffusion dynamics and spatial reorganization of CD1d on the surface membrane of APCs (20). For this reason, we quantitatively compared the spatial characteristics of the actin cytoskeleton in control conditions and ER-stressed THP1 cells. We transduced cells with fluorescently labeled actin (citrine) and studied actin organization in THP1 cells upon ER stress. Visual inspection revealed clear differences in the spatial organization of the actin cytoskeleton in ER-stressed THP1 cells compared to control THP1 cells (Fig. 5*A*). Specifically the total ventral contact area was spatially significantly reduced after treatment with thapsigargin or the SubAB5 toxin (Fig. 5*B*), indicating a compaction of the actin cytoskeleton (43). Unfortunately, we were unable to directly determine actin meshwork sizes from such images, even employing superresolution microscopy approaches, such as STED microscopy, since even their spatial resolution was insufficient to accurately visualize and analyze the 15- to 20-nm small actin mesh sizes.

Next, to understand the molecular processes underlying the decreased contact area, we quantified the turnover dynamics of the actin contact. Actin filaments constantly grow on one end and disassemble on the other by addition and release of monomeric G-actin. This phenomenon can be measured by performing fluorescence recovery after photobleaching (FRAP) measurements on fluorescently tagged monomeric G-actin (44). The G-actin turnover dynamics of the actin filaments were measured by recovery after photobleaching of fluorescence signal in a region of interest. Fig. 5 *C* and *D* display representative fluorescence recoveries of fluorescently tagged G-actin turnover dynamics in thapsigargin and SubAB5 toxin-treated cells compared to untreated control cells. Two characteristics become obvious for ER-stressed cells: 1) A global slow-down in recovery and thus actin turnover dynamics, and 2) an increased fraction of nonrecovered signal (i.e., nonturnovered actin). Both characteristics indicated a nanoscale lengthening of the ventral cortical actin filaments (43), and thus a significant reshaping and tightening of the actin cortex following ER stress.

These results suggest that ER stress not only alters the repertoire of endogenous activating self-lipid antigens, but by inducing CD1d reorganization, it also increases the functional avidity of the CD1d:iNKT–TCR interaction, to further boost the activation of iNKT cells.

### ER-Stressed DCs Activate iNKT Cells In Vivo.

**Fig. 6.**
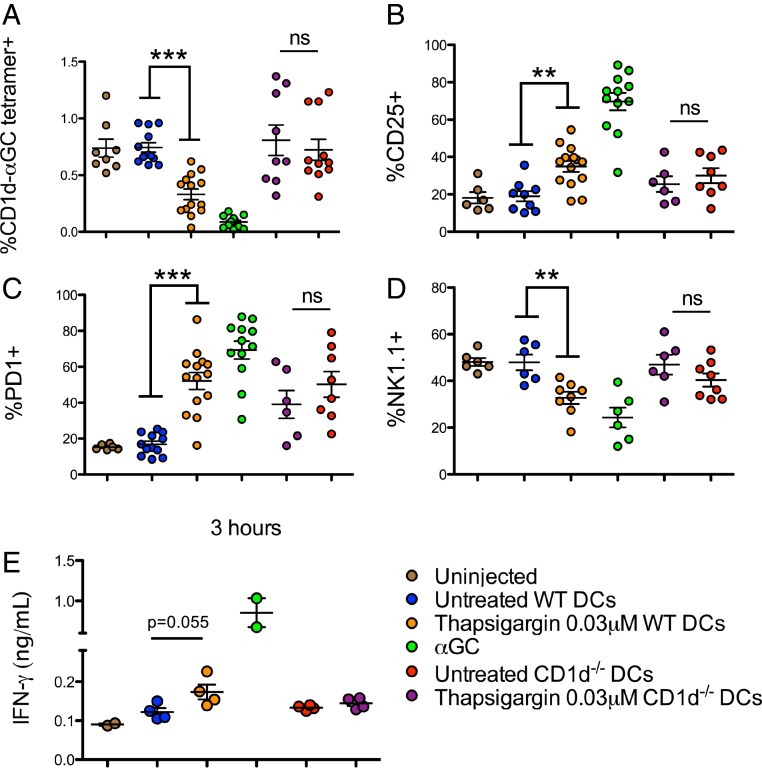
ER-stressed DCs activate iNKT cells in vivo. iNKT cell frequency (*A*) and phenotype (*B*–*D*) in spleens isolated from C57BL/6 mice, 24 h after injection of untreated or thapsigargin treated wild-type or CD1d^−/−^ CD11c^+^ BMDCs. (*E*) IFN-γ levels in the serum of recipient mice at 3 h. **P < 0.005 and ***P < 0.001 by an unpaired, 2-tailed *t* test; ns, not significant. Each dot represents a recipient mouse; animals collated from 3 independent experiments for *A*–*D* and 1 independent experiment for *E*.

To determine whether this mechanism of iNKT cell activation in sterile inflammation can be extended to in vivo settings, CD11c^+^ BMDCs from wild-type and CD1d^−/−^ C57BL/6 mice were treated or not with thapsigargin for 6 h, and then injected intravenously into wild-type C57BL/6 recipient mice. Twenty-four hours after injection, mice were killed and splenic iNKT cell activation was investigated. In mice injected with wild-type thapsigargin-pretreated BMDCs, but not those injected with untreated BMDCs, we observed a decreased percentage of CD1d-αGC tetramer^+^ cells due to TCR down-regulation upon iNKT cell activation (Fig. 6*A*). Consistent with these findings, we also observed increases in the proportion of CD25^+^ (Fig. 6*B*) and PD1^+^ (Fig. 6*C*) iNKT cell populations and decrease in NK1.1 expression on the iNKT cells (Fig. 6*D*). As a control, we observed no significant difference between iNKT cell populations in mice that received untreated or thapsigargin pretreated CD1d^−/−^ DCs. Furthermore, activation markers in conventional CD4^+^ T cells were unchanged in the recipient mice (*SI Appendix*, Fig. S4*D*). To ensure that the thapsigargin-treatment did not influence in vivo DC migration, thapsigargin or untreated BMDCs from CD45.1 and CD45.1/2 mice were injected intravenously into CD45.2 CD1d^−/−^ mice. The injected cells were identified and quantified in the spleen and lungs of recipient mice. We observed that a similar proportion of untreated and thapsigargin-treated DCs migrated to both the spleen and the lungs of recipient mice, although as expected more cells were recovered in the lungs than in the spleen (*SI Appendix*, Fig. S4*E*). An early increase in IFN-γ in the serum of mice injected with thapsigargin pretreated wild-type, but not CD1d^−/−^ DCs further supported in vivo iNKT cell activation by ER-stressed APC (Fig. 6*E*). Together, these results suggest that ER-stressed APC-mediated iNKT cell activation holds true in an in vivo setting.

### ER Stress Is Detected in Both Tumor Cells and Immune Cells in the Tumor Microenvironment.

**Fig. 7.**
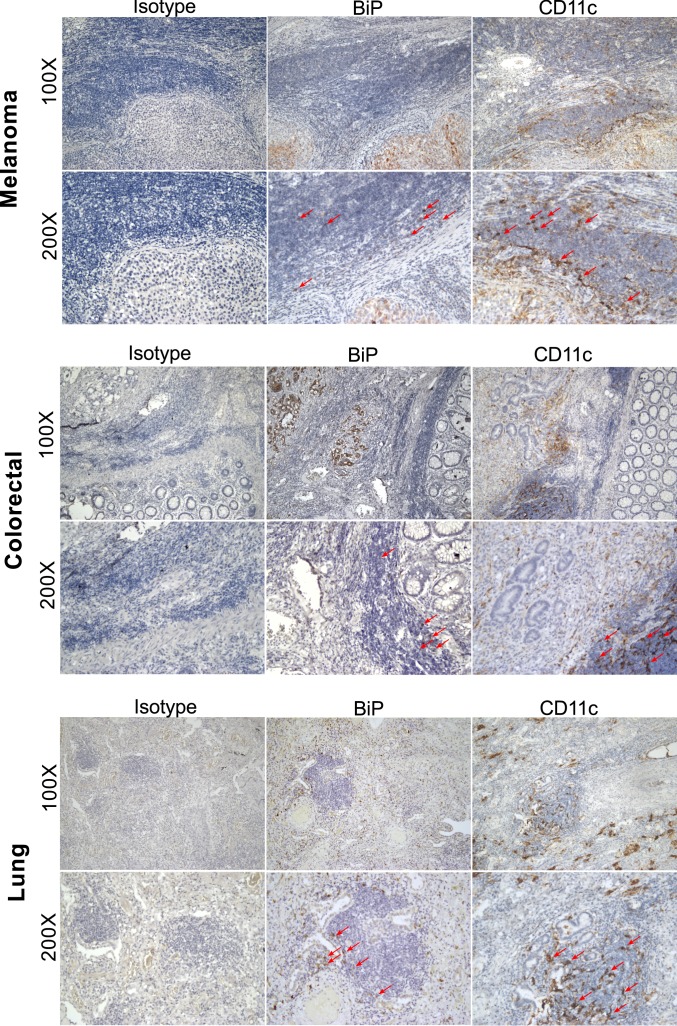
ER stress is detected in both tumor cells and immune cells in the tumor microenvironment. Sequential sections of paraffin-embedded human tumor tissue from patients with melanoma (*Top*), colorectal (*Middle*) and lung (*Bottom*) cancer were stained for BiP and CD11c (brown), with an additional staining for the corresponding isotype controls. Arrows indicate immune cell infiltrate with positive staining for the given marker.

While numerous human “liquid” tumors are known to be CD1d^+^, few solid tumors are reported to express CD1d molecules (45, 46). However, since infiltrating immune cells—particularly CD1d^+^ myeloid cells—would enter a tumor microenvironment wrought with ER stress, they could become ER-stressed themselves (47) and then present immunogenic self-lipids on CD1d to iNKT cells perhaps in the tumor microenvironment or in the draining lymph node. Alternatively, they could cross-present lipids acquired from dying tumor cells (48). To illustrate this possibility, we stained sequential sections from a variety of human solid tumors, including melanoma, colorectal, and lung cancer, for the ER-stress marker BiP and the myeloid cell marker CD11c^+^ (Fig. 7). We observed BiP staining predominantly in the tumor tissue, but also in a number of infiltrating immune cells. Furthermore, some of the infiltrating immune cells are CD11c^+^, suggesting that they could be APCs. Together, we illustrate a biologically relevant setting in human sterile disease where our mechanism of ER stress-mediated iNKT cell activation might hold true.

## Discussion

The results of our experiments identify a previously unknown mechanism by which ER-stressed APCs enhanced sterile activation of iNKT cells both in vitro and in vivo. We showed that during ER stress, PERK-dependent signaling results in the presentation of endogenous lipid agonists on CD1d molecules, which are recognized by iNKT cells. We have extended these results by characterizing the lipid fractions recognized by iNKT cells using recombinant CD1d plate-bound assays. Furthermore, we showed that ER stress alters the spatial surface organization and interaction dynamics of CD1d molecules to modulate CD1d functional avidity. These results highlight a mechanism of iNKT cell activation during sterile conditions of importance both in health and disease, and fill a knowledge gap in our understanding of iNKT cell activation during sterile inflammation, particularly in cancer, where iNKT cells are involved in tumor immunosurveillance (12, 49, 50).

Our results indicated that the PERK branch of the UPR drives the altered self-lipid presentation on APCs under ER stress. Phosphorylation of eIF2α is a necessary step for the self-lipid antigens to be presented, but the subsequent steps remain unclear. However, we demonstrated that the transcription factor ATF4, commonly associated with stress responses, is dispensable for the activation of iNKT cells. Harding et al. (51) have demonstrated that some stress-response genes are regulated in a PERK-dependent, ATF4-independent manner, thus we cannot ignore the possibility that our PERK-dependent, ATF4-indepedent phenotype can be mediated by additional downstream transcriptional events. It is important to also note that the phosphorylation of eIF2α results in selective translation inhibition. Phosphorylation of eIF2α is a convergent step a variety of cellular response pathways collectively termed the integrated stress response. Along with PERK, the general control nonderepressable 2 pathway (GCN2), activated by amino acid deprivation and a parallel input into the integrated stress response (52), might contribute to sterile activation of iNKT cells in certain physiological settings and will be the focus of further investigations.

We also considered autophagy as an additional stress-related pathway that might influence ER stress-mediated iNKT cell activation. Components of the PERK pathway feed into the autophagic process during prolonged stress responses (53) and given that CD1d-loading of the ER stress-mediated self-lipid antigens occurs in lysosome and recycling endosome, they might enter the lysosome through the formation of autophagolysosomes, as might occur during ER-phagy or mitophagy. However, we showed using ATG7^−/−^ BMDCs that autophagy is dispensable in thapsigargin-mediated iNKT cell activation. This finding is consistent with the report by Ganley et al. (54), which illustrates that thapsigargin blocks autophagy by inhibiting formation of autophagosomes.

Numerous studies have focused on the role of ER stress in the tumor microenvironment. Work from the Cubillos-Ruiz and Glimcher groups has investigated the role of XBP1, the main transcription factor downstream the IRE1α pathway, in tumor-infiltrating DCs and T cells. Their studies using CD11c-Cre XBP1^fl/fl^ mice and CD4-Cre XBP1^fl/fl^ mice suggest that XBP1 signaling promotes ovarian tumorgenesis by compromising the antitumor responses of DCs and T cells (55, 56). However, given that XBP1-dependent transcription largely mediates recovery from the misfolded protein burden, in the absence of this branch, there could in fact be enhanced ER stress (57, 58), and dominated by signaling through the other 2 branches, PERK and ATF6α. The enhanced PERK signaling in the XBP1 knockout DCs might contribute to the improved antitumor responses compared to wild-type DCs, through sterile activation of iNKT cells.

Our results support the hypothesis that ER-stressed APCs present alternative self-lipid antigens on CD1d that drive iNKT cell activation. However, it remains unclear whether the lipids involved in ER stress-mediated iNKT cell activation are generated de novo or are preexisting self-lipids that are preferentially loaded onto CD1d. It is known that enzymes involved in their production, through catabolism of longer lipids, or in modification of structural features of the lipid, might also be altered by a changing transcriptional and translational profile in ER-stressed cells. This hypothesis is consistent with the transcriptional down-regulation of enzymes that degrade α-anomeric ceramide-based lipids in ER-stressed DCs (33, 59). Furthermore, XBP1-mediated transcription has been shown to drive expression of lipogenic enzymes in hepatocytes, linking ER stress to modulated lipogenesis (60). We also cannot rule out the possibility that changes in the CD1d-loading machinery under ER-stress conditions might contribute to the preferential loading of immunogenic self-lipid antigens. Microsomal triglyceride transfer protein (MTP) is involved in CD1d loading of lipids in the ER (61). Its function can be modified when it forms heterodimers with specific PDI, a chaperone up-regulated upon ER stress (62). Independent of PDI, MTP was also shown to regulate lysosome CD1d trafficking (63). These observations point to a potential role for MTP/PDI in loading lipid antigen on CD1d in ER stress-mediated iNKT cell activation.

In addition to our finding that ER-stressed APCs up-regulate CD1d molecules loaded with endogenous lipid agonists for iNKT cells, our STED microscopy results revealed actin-driven altered spatial CD1d reorganization in wild-type ER-stressed cells expressing wild-type and GPI-linked CD1d molecules. Although the PERK pathway has been linked to actin filament formation (64, 65), other UPR pathways might also contribute to changes in the surface distribution of CD1d molecules. Indeed, IRE1α signaling can direct restructuring of the actin cytoskeleton in a manner similar to PERK (66). Additional experiments are warranted to investigate the involvement of the other UPR pathways in this system. While altered cytoskeleton organization might impact ER-stressed DC migration in vivo, thereby affecting iNKT cell activation in certain tissue sites, we found that both unstressed and stressed DCs injected intravenously migrate in similar proportion to the lungs and spleen.

Human solid tumor sections were stained to illustrate the presence of APCs in the ER-stressed tumor microenvironment. Transmissible ER stress between solid tumor cells and myeloid cells has been well defined in previous studies (47, 67, 68), and is even implicated in survival and chemoresistance of certain solid tumors (69). To illustrate our hypothesis, we used antibodies against BiP and CD11c. Since most CD11c^+^ cells would also express CD1d molecules, those cells in the tumor microenvironment might activate iNKT cells either in the tumor itself or in the draining lymph node.

While our findings are focused on the up-regulation of lipids bound to CD1d molecules during ER stress that activate iNKT cells, ER stress might have a broader effect on the activation of group I CD1-restricted T cells. Consistent with this possibility, it is known that endogenous lipid hydrocarbon chains can be incorporated as spacer molecules into the groove of CD1 molecules, enhancing functionality of CD1 binding (70). In addition, our findings might also have important implications for our understanding of thymic iNKT cell development. The identity of the self-lipid that mediates positive selection of iNKT cells remains uncertain. Given that ER stress contributes to the maturation of thymocytes (71), it is possible that ER stress-mediated self-lipid antigens are involved in iNKT cell thymic selection and development.

## Materials and Methods

### Reagents.

For a completed list of reagents, please see *SI Appendix*.

### Cell Culture.

APCs were cultured in supplemented RPMI-1640 culture medium (Sigma). Sorted human T cells were cultured in Iscove’s Modified Dulbecco’s Medium (Sigma) containing recombinant IL-2 (1,000 U/mL) and 5% human serum. CD14^+^ monocytes were supplemented with human IL-4 (500 U/mL) and 50 ng/mL human GM-CSF (Peprotech) upon monocyte isolation and were fully differentiated on day 5. MoDCs pulsed with αGC were cocultured with the autologous CD14^−^ fraction. From this, iNKT cells were sorted and expanded as previously described. BM cells were plated at 2 million cells per well in a 6-well plate. Medium was replenished with fresh GM-CSF (20 ng/mL) every 2 d for 5 to 7 d for differentiation into CD11c^+^ BMDCs.

### THP1 Cells Overexpressing Modified CD1d.

THP1 cells overexpressing wild-type and tail^−/−^ CD1d were described previously (72). THP1 cells overexpressing GPI-linked CD1d were made by transducing cells with the CD1d lentiviral vector containing the GPI sequence used in the construct, as previously described (73).

### XBP1 PCR.

RNA was extracted from cell lysates using the RNeasy Mini Kit (Qiagen) following the manufacturer’s instructions. cDNA was synthesized using the High-Capacity Reverse Transcription kit (Applied Biosystems) following the manufacturer’s instructions. XBP1 mRNA was amplified from the cDNA by PCR using the OneTaq 2× Master Mix with Standard Buffer (New England Biolabs) following the manufacturer’s instructions. The primers were purchased from Sigma, based on previously published sequences (*SI Appendix*).

### Quantitative RT-PCR.

Assuming 100% efficiency of the reverse-transcriptase PCR, 10 ng of cDNA in 5-μL nuclease free water was loaded per well of a qPCR plate (MicroAMP Fast Optical 96-well Reaction Plate with Bardcode [0.01 mL]). TaqMan probes using the FAM reporter system (Applied Biosystems by Life Technology) were diluted 20× into TaqMan Fast Advanced Master Mix (Thermo Fisher Scientific) and 5 μL were loaded into each well. The plates were read using the QuantStudio7 (Life Technology).

### iNKT-TCR Tetramer Assay.

Biotinylated NKT-TCR monomer was tetramerized with streptavidin conjugated to PECF 594 (PE-Dazzle, Biolegend), using 12 μg of streptavidin for 50 μg of monomer. The cells were stained using 0.1 μL per well for 40 min on ice, based on a previously described protocol (16).

### CD1d Plate-Bound Assay.

The CD1d plate bound assay was performed as previously described (74). Lipid fractions were added, in duplicate, in 50 μL 50 mM citrate-phosphate buffer pH5-6 and incubated overnight.

### Generation of IRE1, PERK, ATF6α, ATF4 shRNA KD THP-1 Cells.

First, 20,000 wild-type THP-1 cells in 100 μL were plated in a 48-well plate. Then, 50,000 shRNA-loaded pKLO.1 lentiviral particles (Mission shRNA, Sigma) were added to the cells in 100 μL of medium (multiplicity of infection 2.5). Cells with expanded and maintained in R-10 with 2 μg/mL puromycin. KD was confirmed by Western blot.

### Lipid Isolation and Fractionation.

Approximately 25 × 10^6^ THP1 wild-type or PERK KD cells were treated as previously described. They were pelleted and snap frozen in dry ice. Pellets were taken up in 0.9 mL 1:10 PBS: MilliQ water and subjected to 3 freeze–thaw cycles. Lipids were extracted using a 2:1 chloroform:methanol solution. Six fractions containing different lipid classes were then eluted sequentially using amino-propyl columns as described in *SI Appendix*.

### Lipid Quantification by Shotgun MS.

For lipid quantification by shotgun MS, 700 μL of a mixture of internal standards in MTBE/MeOH (5:1.5; [vol/vol]) were added to the dried lipid fractions. MS analyses were performed as previously described (74) on a Q Exactive instrument (Thermo Fisher Scientific) equipped with a robotic nanoflow ion source TriVersa NanoMate (Advion BioSciences) using nanoelectrospray chips with the diameter of spraying nozzles of 4.1 μm. The ion source was controlled by the Chipsoft 8.3.1 software (Advion BioSciences). Spectra was filtered based on repetition rate as previously described and analyzed by a laboratory-developed script (75, 76). Lipids were identified by LipidXplorer software (77).

### Cell Staining, Microscopy, and CD1d Diffusion and Spatial Autocorrelation Analysis.

THP1-CD1d cells were labeled with fluorescently conjugated CD1d (51.1) or ICAM1 Fabs and monomeric soluble iNKT-TCR. For CD1d diffusion experiments, sFCS microscopy in combination with FoCuS-scan software (*SI Appendix*) was used to measure the transit time of CD1d molecules across a designated point on the cell surface. Confocal and STED microscopy was used to image cells stained with the iNKT-TCR monomer and ICAM1 Fab, respectively. Spatial autocorrelation analysis was performed on THP1-CD1d cells stained with the CD1d Fab and imaged with STED microscopy.

### FRAP and Actin Distribution Analysis.

THP-1 CD1d (G-actin citrine) cells under different treatment conditions were analyzed for actin polymerization dynamics using FRAP (44), and were used for taking microscopic images of fluorescent actin. Quantification was performed using Fiji. The quantified area for each cell in different samples were then compared using GraphPad Prism 7 using 2-tailed unpaired *t* test with Welch’s correction (****P < 0.0001).

### In Vivo.

Animal studies were performed with appropriate United Kingdom Home Office licenses, with ethical approval from the University of Oxford. C57BL/6 wild-type and CD1d^−/−^ (C57BL/6-*Cd1d1*^*tm1.2Aben*^/J, JAX stock # 017294) were cared for at the Biomedical Service Unit (John Radcliffe Hospital, Oxford, United Kingdom). Intravenous tail-vein injections were performed using 500,000 or 1 × 10^6^ ex vivo differentiated BMDCs. Spleens and lungs were harvested, processed into a single-cell suspension (lungs using 0.75 mg/mL DNase and 0.5 mg/mL collagenase at 37**°**C for 1 h), and treated with red blood cell lysis buffer before being stained for flow cytometry analysis. Blood was obtained from recipient mice via tail-bleeding let to clot at room temperature for 30 min, and spun at 4,000 × *g* for 15 min to separate the serum used in the ELISA.

### Immunohistochemistry.

Slides of paraffin-embedded tissue sections were provided by Oxford Centre for Histopathological Research under the project code 19/A075 and 16/A194. Staining was performed following the manufacturer’s instructions (Dako, Cell Signaling).

### Statistical Analysis.

Statistical analysis was performed where biological replicates *n* ≥ 3. IFN-γ secretion was assumed to follow a Gaussian distribution. Points represent the mean of technical duplicates for each biological experiment, and the error bars represent SEM. Statistical analysis was performed in Graphpad Prism v5.0a.

## Supplementary Material

Supplementary File
