# Supplementary material for: Sterile activation of invariant natural killer T cells by ER-stressed antigen-presenting cells
Source: Proc Natl Acad Sci U S A. 2019 Nov 5;116(47):23671–81. doi: 10.1073/pnas.1910097116 (PMC6876220; doi:10.1073/pnas.1910097116)
Supplement: Supplementary File [file pnas.1910097116.sapp.pdf]

Supplementary information for:

Sterile activation of invariant Natural Killer T cells by ER-stressed antigen presenting cells

Melissa Bedard[a], Dilip Shrestha[a], David A. Priestman[b], Yuting Wang[c],[d], Falk Schneider[a], Juan D. Matute[e,f], Shankar S. Iyer[e], Uzi Gileadi[a], Gennaro Prota[a], Matheswaran Kandasamy[a], Natacha Veerapen[g], Gurdyal Besra[g], Marco Fritzsche[a,h], Sebastian Zeissig[c,i], Andrej Shevchenko[d], John C. Christianson[j], Frances M. Platt[b], Christian Eggeling[a,k,l], Richard S. Blumberg[e], Mariolina Salio[a], and Vincenzo Cerundolo[a,1]

[a]Medical Research Council Human Immunology Unit, Weatherall Institute of Molecular Medicine, University of Oxford, OX3 9DS Oxford, United Kingdom; [b]Department of Pharmacology, University of Oxford, OX1 3QT Oxford, United Kingdom; [c]Center for Regenerative Therapies, Technische Universität Dresden, 01307 Dresden, Germany; [d]Max Planck Institute of Molecular Cell Biology and Genetics, 01307 Dresden, Germany; [e]Division of Gastroenterology, Department of Medicine, Brigham and Women's Hospital HarvardMedical School, Boston,MA 02115; [f]Division of Neonatology, Department of Pediatrics, Massachusetts General Hospital, Harvard Medical School, Boston, MA 02114; [g]School of Biosciences, University of Birmingham, B15 2TT Egdbaston, United Kingdom; [h]Kennedy Institute for Rheumatology, University of Oxford, OX3 7LF Oxford, United Kingdom; [i]Department of Medicine I, University Medical Center Dresden, Technische Universität Dresden, 01307 Dresden, Germany; [j]Botnar Research Centre, Nuffield Department of Orthopaedics, Rheumatology, and Musculoskeletal Science, University of Oxford, OX3 7LD Oxford, United Kingdom; [k]Institute of Applied Optics and Biophysics, 07743 Jena, Germany; and [l]Department of Biophysical Imaging, Leibniz Institute of Photonic Technologies e.V., 07745 Jena, Germany

This PDF includes:

Supplementary text
Figures S1 to S5
Legends for Figures S1 to S5
SI References

www.pnas.org/cgi/doi/10.1073/pnas.1910097116

Supplementary Information Text

Materials and Methods

Reagents

Antibodies for flow cytometry included anti-human: CD3-APC clone HIT3a (BD Biosciences), CD4-FITC clone RPAT4 (BD Biosciences), ICAM1-PE clone HA58 (Biolegend), CD25-APC and –FITC clone M-A251 (BD Biosciences), CD25-PerCP-Cy5.5 (BC96, Biolegend) and CD1d-PE clone 42.1 (BD Biosciences). Dead cells were gated out using LIVE/DEAD Fixable Aqua Dead Cell Stain Kit (Life Technologies) or propidium iodide solution (Sigma-Aldrich). Anti-mouse antibodies were purchased from eBiosciences (PD1-APC clone J43, CD44-Alexa700 clone IM7, CD69-PECy7 clone H1.2F3, CD16/32 clone 93) or Biolegend (NK1.1-Alexa488 clone PK136, CD25-BV650 clone PC61, B220-BV510 clone RA3-6B2, CD8α-BV711clone 53-6.7) Thapsigargin (stock concentration 1mM in DMSO), tunicamycin (stock concentration 1mg/mL in DMSO), and DMSO were all purchased from Sigma. Antibodies for western blotting included: PDI (C81H6, Cell Signaling), BiP (C50B12, Cell Signaling), CHOP (D46F1, Cell Signaling), ATF4 (D4B8, Cell Signaling), PERK (ab65124, Abcam) and GADPH (sc-365062, Santa Cruz; 5174S, Cell Signaling). CD11c (clone EP134Y, ab52632) and the matched isotype control (Mouse IgG1κ, ab170190) and the previously described BiP antibody and the matched isotype control (Rabbit IgG, 3900S), were used for immunohistochemistry. LEAF Purified anti-human CD1d (clone 51.1 from Biolegend) and the corresponding isotype control LEAF Purified Mouse IgG2bκ were used in the CD1d blocking assays. Similarly, the IL12/IL23p40 (clone C8.6) blocking antibody and isotype control was purchased from Biolegend. For human IFN-γ and human IL12p40 ELISA, the purified coating and biotin-conjugate detection antibodies were all purchased from BD Pharmingen. Protein standards were purchased from Peprotech. α-galactosylceramide was either synthesized by Gurdyal Besra or acquired commercially (KRN7000, Enzo Life Sciences). 5ARU was synthesized in house

(1). (E)-1-Hydroxy-2-methyl-2-butenyl 4-pyrop (HMBPP) was purchased from Sigma Alrich and resuspended as per manufacturer instructions. GSK2606414 (Sigma) ISRIB (SML0843), MKC3964 (Calbiochem), STF-083010 (SML0409) and Ceapin-A7 (SML2330) were purchased from Sigma Aldrich and resuspended as per manufacturer instructions. The SubAB5 toxin was generously gifted by Drs. John Christianson (University of Oxford) and James Paton (University of Adelaide).

Cell Culture

WT THP1 and THP1 CD1d cell lines, differentiating BMDCs, differentiating human MoDCs were cultured in complete medium: RPMI-1640 culture medium (Sigma) containing 10% heat inactivated FCS (Gibco), 2mM L-glutamine (Sigma), pencillin-streptomycin 100X (Sigma), 1mM sodium pyruvate (Gibco), MEM non essential amino acids 100X (Gibco), 10mM HEPES buffer solution (Gibco), 50uM β-mercaptoethanol (Gibco). Sorted human iNKT cells were cultured in Iscove's Modified Dulbecco's Medium (Sigma) containing recombinant IL-2 (1000U/mL) and 5% human serum. $CD14^+$ monocytes were supplemented with human IL-4 (500U/mL) and 50ng/mL human GM-CSF (Peprotech) upon monocyte isolation and were fully differentiated on day 5.

Isolation of $CD14^+$ monocytes and iNKT cells

PBMCs were isolated from leukocyte cones (purchased from the NHS blood and transport unit) as previously described. The PBMCs were incubated with $CD14^+$ beads (Miltenyi) and the purified using LS MACS columns (Miltenyi) following the manufacturer's instructions. Purified $CD14^+$ monocytes were differentiated into MoDCs, pulsed with αGC and co-cultured with the autologous $CD14^-$ fraction. From this, iNKT cells were sorted and expanded as previously described.

Isolation of MAIT and γδ T cells

MAIT and γδ cells were sorted from healthy donor PBMCs with the following antibodies: Vα7.2 (clone 3C10), CD161 (clone HP-3G10), γδ (clone B1). All antibodies were from Biolegend.

Isolation of BMDCs

Wild type and CD1d$^{-/-}$ C57BL/6 mice (described below) were sacrificed following regulations. The long bones (femur and tibia) were collected. The bone marrow, flushed using a 19-gauge needle and syringe with complete medium, was passed through a cell strainer to create a single cell suspension. Cells were plated at 2 million cells/well in a 6-well plate. Medium was replenished with fresh GMCSF (20ng/mL) every 2 days for 5-7 days.

THP1 cells overexpressing modified CD1d

THP1 cells overexpressing wild type and tail$^{-/-}$ CD1d were described previously (2). THP1 cells overexpressing GPI-linked CD1d were made by transducing cells with the CD1d lentiviral vector containing the GPI sequence used in the construct as previously described (3).

Flow Cytometry

Flow cytometry samples were run either on the Dako Cytomation CyAn ADP, BD FACSCanto, Fortessa X50, or the Nxt Attune flow cytometers and analyzed by FlowJo software. The gating was on lymphocytes or APCs (based on forward and side scatter), single cells, and live cells, as illustrated in Supplementary Figure 4B. Cells were counted in specific gates using the Nxt Attune software.

ELISA

The ELISA was performed using Costa half area, high binding polystyrene 96 well plates (Corning), and was performed following the manufacturer’s instructions.

Western blotting

Cytosolic protein from cell lysates was quantified using a BCA assay (Thermo Scientific). A constant amount of protein (20μg) was loaded into pre-cast gels (4-12 or 10% polyacrylimide) and run at 120V. The western blotting protocol was performed as per manufacturer's instructions.

XBP1 PCR

RNA was extracted from cell lysates using the RNeasy Mini Kit (Qiagen) following the manufacturer's instructions. cDNA was synthesized using the High-Capacity Reverse Transcription kit (Applied Biosystems) following the manufacturer's instructions. XBP1 mRNA was amplified from the cDNA by PCR using the OneTaq 2X Master Mix with Standard Buffer (New England Biolabs) following the manufacturer's instructions. The forward primer was: 5' - TTACGGGAGAAAACTCACGGC -3'. The reverse primer was: 5'-GGGTCCAACTTGTCCAGAATGC-3'. The primers were purchased from Sigma.

Quantitative RT-PCR

Assuming 100% efficiency of the reverse transcriptase PCR, 10ng of cDNA in 5μL nuclease free water was loaded per well of a qPCR plate (MicroAMP® Fast Optical 96-well Reaction Plate with Barcode (0.01mL)). TaqMan probes using the FAM reporter system (Applied Biosystems by Life Technology) were diluted 20X into TaqMan Fast Advanced Master Mix (Thermo Fisher Scientific) and 5 μL were loaded into each well. The plates were read using the QuantStudio7 (Life Technology).

iNKT-TCR tetramer assay

Biotinylated NKT-TCR monomer was tetramerized with streptavidin conjugated to PECF 594 (PE-Dazzle, Biolegend), using 12μg of streptavidin for 50μg of monomer. The cells were stained using 0.1μL/well for 40 minutes on ice, based on a previously described protocol (16).

CD1d plate bound assay

The CD1d plate bound assay was performed as previously described (77). Lipid fractions were added, in duplicate, in 50uL 50mM citrate-phosphate buffer pH5-6 and incubated overnight.

Generation of IRE1, PERK, ATF6α, ATF4 shRNA knock down THP-1 cells

20,000 wild type THP-1 cells in 100μL were plated in a 48-well plate. 50,000 shRNA–loaded pKLO.1 lentiviral particles (Mission shRNA, Sigma) were added to the cells in 100μL of medium (MOI 2.5). Cells with expanded and maintained in R-10 with 2μg/mL puromycin. Knock down was confirmed by western blot.

Lipid Isolation and Fractionation

Approximately $25X10^6$ THP1 WT or PERK KD cells were treated for six hours with thapsigargin 0.03μM or left untreated. The cells were washed and left to incubate overnight, after which they were pelleted and snap frozen in dry ice. Pellets were taken up in 0.9 mL 1:10 PBS: MilliQ water and subjected to 3 freeze-thaw cycles. Chloroform (1.5 mL) and methanol (1.5 mL) was added and centrifuged 3,000 rpm for 10 minutes to remove insoluble/precipitated protein. 0.5 mL PBS was added and the lower phase, containing lipids of interest, removed to a new tube, dried down under a stream of nitrogen and resuspended in 1mL chloroform. Amino-propyl columns (SUPELCO Superclean LC-NH2, 1 mL) were pre-equilibrated with 3mL n-hexane. Lipids in chloroform applied to the column and 1mL chloroform used to rinse the lipid sample tube and also applied to the column. The unbound fraction was retained. Six fractions containing different lipid classes were then eluted sequentially as follows: Fraction 1 (containing cholesterol, diglycerides and tri-glycerides) was eluted with 4 mL diethylether; Fraction 2 (ceramides and monoglycerides): 3 mL chloroform/ methanol 23:1 (v/v); Fraction 3 (free fatty acids and free hydroxy-fatty acids): 2 mL di-isopropyl ether/acetic acid (98/4); Fraction 4 (neutral glycolipids and sphingoid bases): 2mL acetone/methanol 9:1.2; Fraction 5 (neutral

phospholipids, phosphatidylcholine, phosphatidylethanolamine and sphingomyelin): 2mL chloroform/methanol 2:1. The fractions were then dried down under a stream of nitrogen and retained for further analyses.

Chemicals and lipid standards for mass spectrometry analysis

Common chemicals and solvents were LC-MS grade from Sigma–Aldrich Chemie (Munich, Germany) and methanol (LiChrosolv grade) from Merck (Darmstadt, Germany). Synthetic lipid standards were purchased from Avanti Polar Lipids, Inc. (Alabaster, AL, USA). Internal standard mix was prepared in methyl-tert-butyl ether (MTBE)/methanol (MeOH) (5:1.5; v/v) containing 1299.73 pmol of cholesterol ester (CE)-D7 16:0; 2133.62 pmol of cholesterol (Chol)-D7; 1457.65 pmol of triacylglycerol (TG)-D5 50:0; 145.35 pmol of diacylglycerol (DG)-D5 34:0; 1100.88 pmol of phosphatidylcholine (PC) 25:0; 692.61 pmol of phosphatidylethanolamine (PE) 25:0; 383.62 pmol of phosphatidylinositol (PI) 25:0; 334.07 pmol of phosphatidylserine (PS) 25:0; 77.06 pmol of phosphatidic acid (PA) 25:0; 68.17 pmol of phosphatidylglycerol (PG) 163.21 pmol of lyso-phosphatidylcholine (LPC) 13:0; 106.33 pmol of lyso-phosphatidylethanolamine (LPE) 13:0; 140.63 pmol of lyso-phosphatidylinositol (LPI) 13:0; 113.51 pmol lyso-phosphatidic acid (LPA) 13:0, 272.42 pmol of ceramide (Cer) 30:1:2; 554.90 pmol of sphingomyelin (SM) 30:1:2 and 135.88 pmol of galactosylceramide (GalCer) 30:1:2 and stored at −20 °C until the analysis. The annotation of lipid species is according as previously described (4). Glycerolipid and glycerophospholipid species were annotated by the number of carbon atoms: double bonds in all fatty acid moieties. Sphingolipid species were annotated by the number of carbon atoms:double bonds:hydroxyl groups at the ceramide backbone.

Lipid quantification by shotgun mass spectrometry

700 µl of a mixture of internal standards in MTE/MeOH (5:1.5; v/v) were added to the dried lipid fractions. Mass spectrometric analyses were performed as previously described (5) on a Q Exactive instrument (Thermo Fisher Scientific,

Bremen, Germany) equipped with a robotic nanoflow ion source TriVersa NanoMate (Advion BioSciences, Ithaca NY, USA) using nanoelectrospray chips with the diameter of spraying nozzles of 4.1 µm. The ion source was controlled by the Chipsoft 8.3.1 software (Advion BioSciences). Spectra was filtered based on repetition rate as previously described and analyzed by a lab-developed script (6, 7). Lipids were identified by LipidXplorer software (8).

Cell labeling for live cell sFCS diffusion measurements

Antibodies against CD1d were produced and affinity-purified from the hybridoma 51.1.3 that has been previously described (9). The cell line was a gift from Prof. Steve Porcelli (Albert Einstein College, New York). For sFCS experiments, cells were first washed twice with L15 media. Then, they were incubated with ~10 µg/ml of Alexa 488 Fabs in 50µL of L15 for 10 min at room temperature. Next, cells were washed in L15 thoroughly and were dropped on a 25 mm ethanol-cleaned coverslips. Cells were allowed to attach on coverslips for ~15min before sFCS data were recorded. All the experiments were carried out at 37°C in L15 medium.

Cell labeling for determining plasma membrane CD1d distribution and iNKT-TCR staining

THP1 CD1d cells were washed first and were resuspended in L15 and allowed to attach on an 18 mm ethanol-cleaned coverslips assembled in a chamber for 1 h at 37ºC. They were subsequently labeled with Abberior STAR RED-51.1.3 Fabs (~10µg/ml) for 10min at room temperature. Following Fab labeling, cells were washed twice with L15 and with cytoskeleton buffer before being fixed with 4% paraformaldehyde (PFA) and 0.1% glutaraldehyde (GA) in cytoskeleton buffer for 15 min at room temperature. The sample was then washed and kept in cytoskeleton buffer until measurements were done in the microscope. A mixture of 20nM biotin-conjugated iNKT-TCR monomer and 1µM D-biotin was prepared and 20nM AbbStar 635p Neutravidin was added to label the monomer in PBS, which was finally diluted in L15. THP1-CD1d cells in IBIDI chambers were

stained with this final mixture for 10min at room temperature, washed with PBS, fixed with 4% paraformaldehyde (PFA) and 0.2% glutaraldehyde (GA), then imaged.

Scanning FCS Measurement

A commercial Leica TCS SP8 STED 3X microscope (Leica Microsystems GmBH, Mannheim, Germany) was used for sFCS experiments. The instrument is supplied with a pulsed super-continuum white-light laser (WLL, NKT Photonics, 80 MHz) making it possible to select any wavelength of choice for the excitation of fluorophores in the entire visible spectrum. The emitted fluorescence was collected using a hybrid single molecule detector (SMD2) in the range of 495-585 nm with a detector gain set to 200 respectively. This equates to 2.15μs pixel dwell time. The specific regions in the cells were selected by adjusting the zoom size 20X resulting in an area of 5.82×5.82 μm. In most cases, sFCS measurements were performed twice in different parts of the same cell and recordings were done at 37°C to match the physiological conditions.

Analysis of sFCS data

Recently, we published a paper on software dedicated for the analysis of sFCS data called FoCuS-scan (10). This software was used to perform autocorrelation analysis. For our experiments, we restricted our autocorrelation analysis for 10-20s. The initial few seconds of the intensity carpets that included substantial photobleaching were cropped-out. The obtained autocorrelation curve was generated based on a previous publication (11). Therefore, all 'τ_D' values were converted to their ln values and the frequencies were normalized to 1 with the maximum counts being 100%. The two ln distributions were then compared using two-tailed unpaired t-test with Welch's correction with p-value being reported with a statistical significance of <0.0001. The ln (transit time, ms) was also fitted with the non-linear regression Gaussian fitting algorithm of the GraphPad Prism 7. The mean +/- SD and confidence interval (CI) values obtained from the fits were reported thereafter (Table 1).

Fluorescence Recovery after Photobleaching

THP1 CD1d (G-actin citrine) cells under different treatment conditions were used for FRAP experiments. The cells were prepared by washing them twice in L15 buffer then releasing them slowly in a L15 media containing ethanol-cleaned coverslips. Cells were allowed to attach to the coverslips for ~20 min at 37 °C before proceeding for FRAP data acquisitions. The instrumental settings for FRAP data acquisition were based on a previous publication (12). The data were exported to OriginPro 9.1 (Origin Labs, USA) for the analysis. The fluorescence recovery data was normalized to 1 with the maximum intensity – mean intensity of the first two recorded images before photobleaching – as 100%. The normalized fluorescence recovery curves were then plotted against time using two-component exponential fitting strategy described (12). The half-life of the fluorescence recovery was calculated as previously described(13). Half-life and fraction of populations of species (mobile or immobile) obtained from each curves were then compared using a two-tailed unpaired t-test and using Mann-Whitney test (*, **, ***, **** being p- values less than 0.05, 0.01, 0.001 and 0.0001 respectively).

Image Analysis : Quantification of Surface Area for Actin distribution

THP1 CD1d (G-actin citrine) cells under different treatment conditions were prepared as above. Thereafter, microscopic images of fluorescent actin were taken. Quantification was performed using Fiji. Quantified area for each cells in different samples were then compared using GraphPad Prism 7 using two-tailed unpaired t-test with Welch's correction (**** is p-value <0.0001).

Spatial Autocorrelation analysis

For spatial autocorrelation analysis, fixed THP1 CD1d cells were prepared as described above. Leica TCS SP8 STED 3x microscope was used for recording STED images using 100x 1.4-oil immersion objective and SMD2 detector - voltage gain 100. The Abberior STAR RED dye was excited with 633 nm

wavelength and the fluorescence signals were collected with the time-gating being applied in the detector in the range of 0. 5-6.5 ns. Images –33.24 × 33.24 µm area– were acquired with ~25 nm pixel sizes with 8 line accumulation and 2 frame averages. The power for the excitation, 633 nm and STED, 775 nm, lasers at the front of the objective were 30 µW and 175 mW yielding full-width-half-maximum of ~53 +/- 5 nm (Mean +/- SD) from the reference Abberior red fluorescent nanoparticles of 40 nm. The following steps were taken to calculate the spatial autocorrelation function, G(Φ), of the image: Background subtraction of the image from the measured mean value of selected 3-4 different regions in the cell-free parts of the image › select peripheral regions with uniform distribution of CD1d (64× 64 pixel area) › run stack FFT ICS jru v1 (Stowers plugin) › run Radial Profile Plot › save the x-y data for non-linear regression fitting (Gaussian) in GraphPad Prism 7. The data is presented as mean +/- SD for each point.

In vivo

Animal studies were performed with appropriate UK Home Office licenses, with ethical approval from the University of Oxford. C57BL/6 wild-type and CD1d$^{-/-}$ (C57BL/6-*Cd1d1*$^{tm1.2Aben}$/J, JAX stock # 017294) were cared for at the Biomedical Service Unit (John Radcliffe Hospital, Oxford, UK). Intravenous tail-vein injections were performed using 500,000 or 1X10^{6} *ex vivo* differentiated bone marrow-derived dendritic cells suspended in 100µL or 150 µL PBS, respectively. Spleens and lungs were harvested, processed into a single cell suspension (lungs using 0.75mg/mL DNAse and 0.5mg/mL collagenase at 37°C for 1 hour), and treated with red blood cell lysis buffer before being stained for flow cytometry analysis. Blood was obtained from recipient mice via tail-bleeding let to clot at room temperature for 30 minutes, and spun at 4000g for 15min to separate the serum used in the ELISA.

Immunohistochemistry

Slides of paraffin embedded tissue sections were provided by Oxford Centre for Histopathological Research (OCHRE) under the project code 19/A075 and 16/A194. Staining was performed following the manufacturer's instructions (Dako, Cell Signaling).

Statistical Analysis

Statistical analysis was performed where biological replicates N were equal to or greater than three. IFN-γ secretion was assumed to follow a Gaussian distribution. Points represent the mean of technical duplicates for each biological experiment, and the error bars represent standard error around the mean. Statistical analysis was performed in Graphpad Prism Version 5.0a.

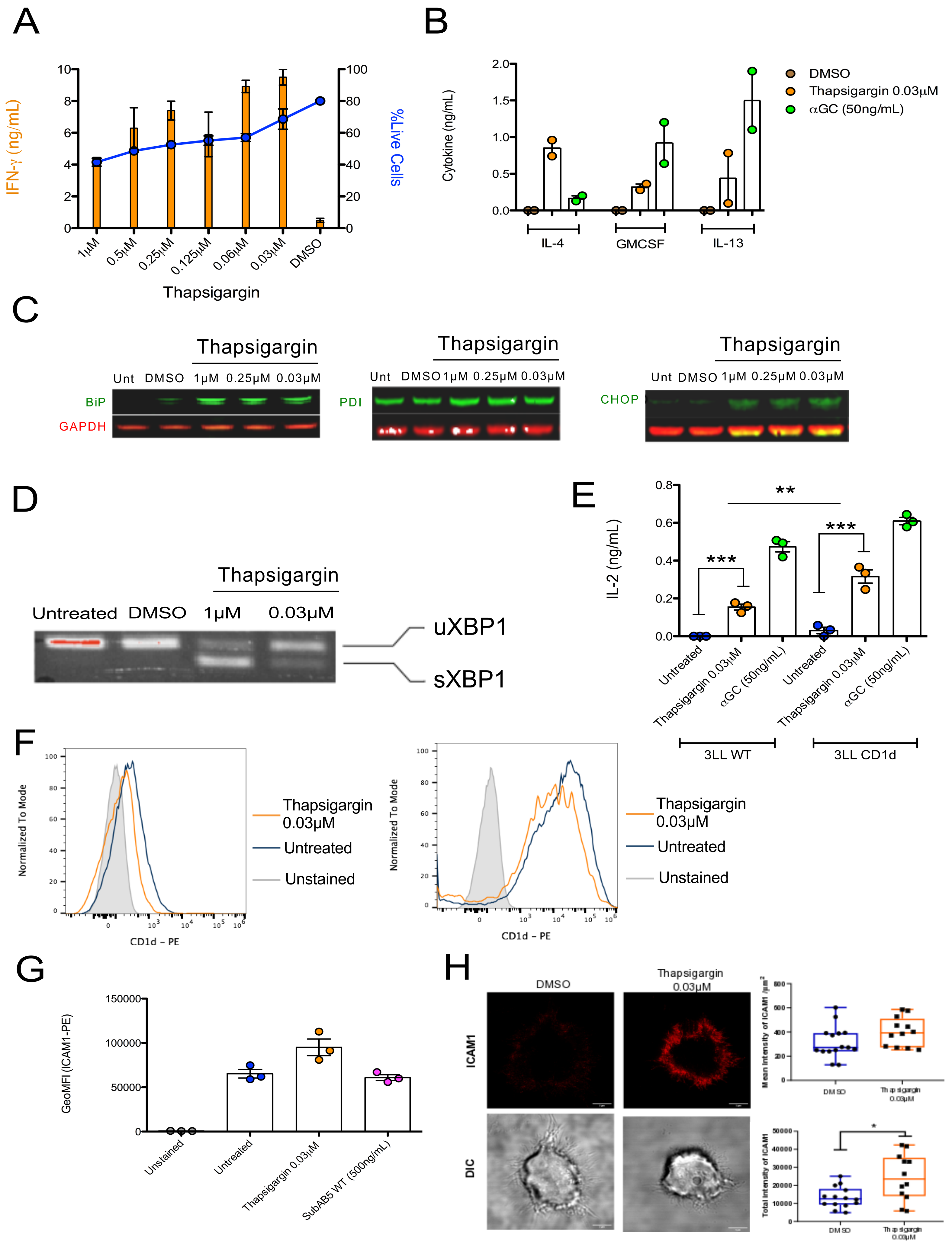

A
IFN-γ (ng/mL)
%Live Cells
1μM
0.5μM
0.25μM
0.125μM
0.06μM
0.03μM
DMSO
Thapsigargin
B
Cytokine (ng/mL)
DMSO
Thapsigargin 0.03μM
αGC (50ng/mL)
IL-4
GMCSF
IL-13
C
Thapsigargin
Unt DMSO 1μM 0.25μM 0.03μM
BiP
GAPDH
PDI
CHOP
D
Thapsigargin
Untreated DMSO 1μM 0.03μM
uXBP1
sXBP1
E
IL-2 (ng/mL)
Untreated
Thapsigargin 0.03μM
αGC (50ng/mL)
3LL WT
3LL CD1d
F
Normalized To Mode
CD1d – PE
Thapsigargin 0.03μM
Untreated
Unstained
G
GeoMFI (ICAM1-PE)
Unstained
Untreated
Thapsigargin 0.03μM
SubAB5 WT (500ng/mL)
H
DMSO
Thapsigargin 0.03μM
ICAM1
DIC
Mean Intensity of ICAM1 /μm²
Total Intensity of ICAM1

Figure S1: Thapsigargin treatment of APCs induces iNKT cell activation and UPR. (A) iNKT cell activation (IFN-γ secretion, left Y axis) and viability of MoDCs (right Y axis) upon treatment with the indicated decreasing concentrations of thapsigargin. Data representative of N=2 biological replicates. **(B)** IL-4, GMCSF, and IL-13 secretion in co-cultures of iNKT with thapsigargin-treated or αGC-pulsed MoDCs. Average of N=2 biological replicates. **(C)** Western blot validation of ER stress and UPR in THP1 cells treated with the indicated concentrations of thapsigargin. Green bands represent protein expression of BiP (left), PDI (center) and CHOP (right); red bands represent loading controls (GAPDH). **(D)** mRNA splicing of the transcription factor XBP1 upon thapsigargin treatment of THP1 cells, indicating increased ER-stress and UPR activation. **(E)** Murine iNKT (hybridoma DN32.2) cell activation by Lewis Lung Carcinoma cells (3LL), either wild type or overexpressing CD1d molecules, thapsigargin-treated or pulsed with αGC ** and *** represents $p<0.005$ and $p<0.001$, respectively, by one-way ANOVA with a Bonferroni post-test. Average of N=3 biological replicates. **(F)** CD1d surface expression on thapsigargin-treated WT or CD1d overexpressing THP1 cells. The histograms are representative of N=2. **(G)** ICAM1 surface expression on thapsigargin or SubAB5-treated MoDCs. Each dot represents a biological replicate of N=3, each performed in technical duplicates. **(H)** ICAM staining in thapsigargin-treated THP1-CD1d cells compared to control treated cells. Left, STED microscopy (FWHM = 95±5nm) and DIC images; right, ICAM mean intensity staining/μm^2 (top) and total intensity (bottom). * indicates $p<0.05$ with two-tailed Mann-Whitney test. Each dot in the box-plot represents corresponding value for one cell.

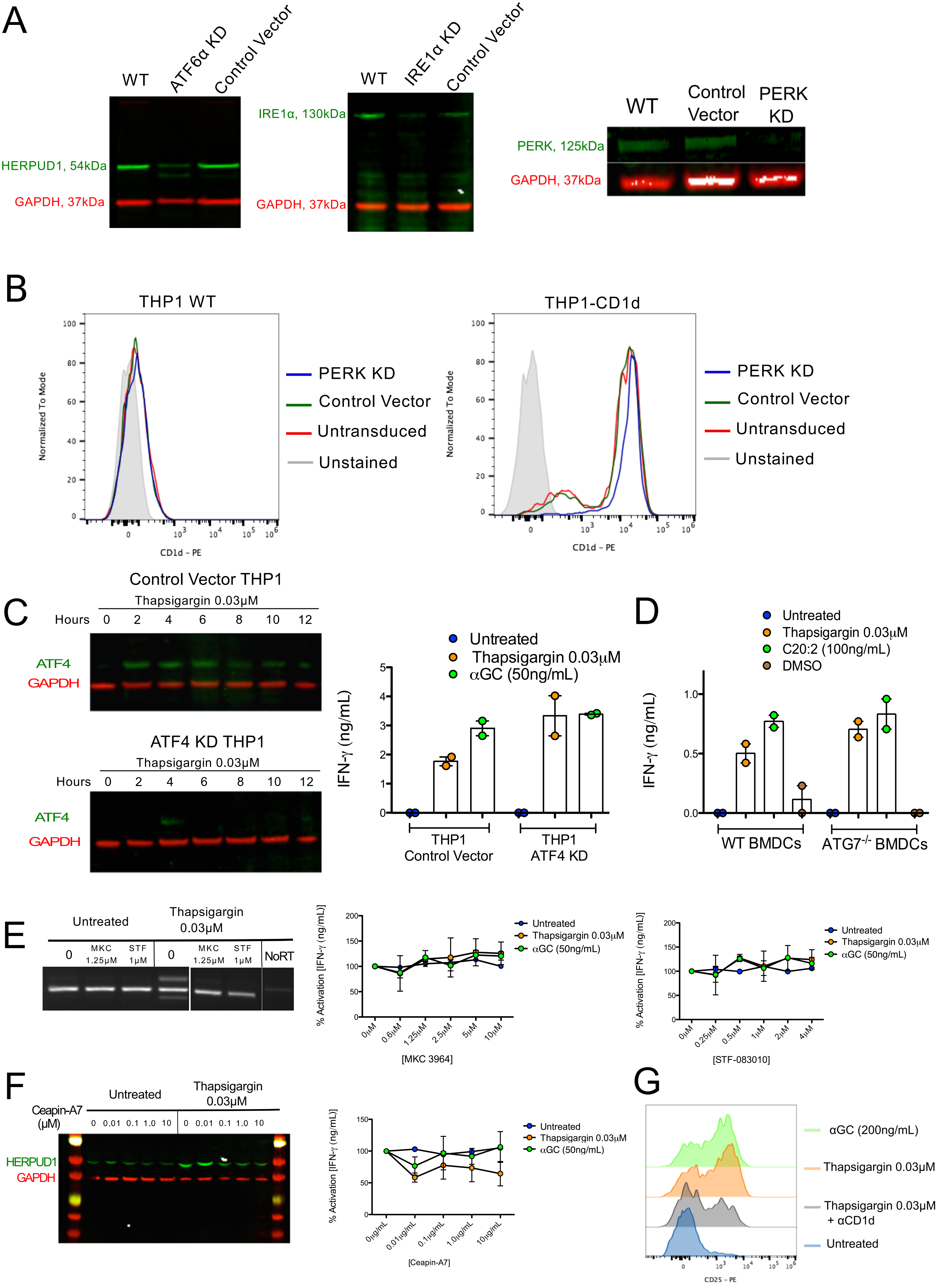
A
WT
ATF6α KD
Control Vector
HERPUD1, 54kDa
GAPDH, 37kDa
WT
IRE1α KD
Control Vector
IRE1α, 130kDa
GAPDH, 37kDa
WT
Control Vector
PERK KD
PERK, 125kDa
GAPDH, 37kDa
B
THP1 WT
THP1-CD1d
Normalized To Mode
CD1d - PE
PERK KD
Control Vector
Untransduced
Unstained
C
Control Vector THP1
Thapsigargin 0.03μM
Hours 0 2 4 6 8 10 12
ATF4
GAPDH
ATF4 KD THP1
Thapsigargin 0.03μM
Hours 0 2 4 6 8 10 12
ATF4
GAPDH
Untreated
Thapsigargin 0.03μM
αGC (50ng/mL)
IFN-γ (ng/mL)
THP1 Control Vector
THP1 ATF4 KD
D
Untreated
Thapsigargin 0.03μM
C20:2 (100ng/mL)
DMSO
IFN-γ (ng/mL)
WT BMDCs
ATG7-/- BMDCs
E
Untreated
Thapsigargin 0.03μM
0
MKC 1.25μM
STF 1μM
0
MKC 1.25μM
STF 1μM
NoRT
% Activation [IFN-γ (ng/mL)]
Untreated
Thapsigargin 0.03μM
αGC (50ng/mL)
0μM 0.6μM 1.25μM 2.5μM 5μM 10μM
[MKC 3964]
0μM 0.25μM 0.5μM 1μM 2μM 4μM
[STF-083010]
F
Ceapin-A7 (μM)
Untreated
Thapsigargin 0.03μM
0 0.01 0.1 1.0 10 0 0.01 0.1 1.0 10
HERPUD1
GAPDH
% Activation [IFN-γ (ng/mL)]
Untreated
Thapsigargin 0.03μM
αGC (50ng/mL)
0μg/mL 0.01μg/mL 0.1μg/mL 1.0μg/mL 10μg/mL
[Ceapin-A7]
G
αGC (200ng/mL)
Thapsigargin 0.03μM
Thapsigargin 0.03μM + αCD1d
Untreated
CD25 - PE

Figure S2: Contribution of the three UPR branches in driving iNKT cell activation in ER-stressed APCs. **(A)** Western blots illustrating the successful lentiviral transduction of THP1 cells with shRNA targeting ATF6α (left), IRE1α (center) or PERK (right). Controls with empty vectors and loading controls (GAPDH) are also shown. **(B)** CD1d surface expression, as measured by flow cytometry, in either untransduced, control vector transduced or PERK KD wild type (left) or CD1d-overexpressing (right) THP1 cells. The histograms are representative of N=3 biological replicates. **(C)** (Left) ATF4 induction upon thapsigargin treatment in control THP1 cells (top), but not in ATF4 knock down cells (bottom). (Right) iNKT cell activation by thapsigargin-treated APCs is independent of ATF4 activity. IFN-γ secretion is the average of N=2 biological replicates. **(D)** iNKT cell activation by thapsigargin-treated BMDCs is independent of ATG7 expression. IFN-γ secretion is the average of N=2 biological replicates. **(E)** iNKT cell activation by thapsigargin treated THP1 cells is independent of IRE1α inhibition. Left panel: the IRE1 inhibitors MKC 3964 and STF-083010 inhibit thapsigargin-induced XBP1 splicing. Centre and right panels: the IRE1 inhibitors MKC 3964 (center) and STF-083010 (right) do not inhibit iNKT cell activation by thapsigargin-treated THP1 cells. IFN-γ secretion is the average of N=2 biological replicates. **(F)** The ATF6α inhibitor Ceapin-A7 reduces expression of the downstream ATF6α target HERPUD1 (left) but does not inhibit iNKT cell activation (right). IFN-γ secretion is the average of N=3 biological replicates. **(G)** Activation of human iNKT cells cultured with lipids from Fraction I with and without a CD1d-blocking antibody (20μg/mL) on the CD1d plate bound assay as measured by CD25 expression by flow cytometry. The histograms are representative of N=2 biological replicates.

A

Summary of sFCS data

Samples		Average Diffusion Time (In values +/-SD)	95% Confidence interval (CI)	*p*- value
DMSO (control)		4.55 +/- 0.64	4.50– 4.60	<0.0001
	Thapsigargin 0.03 µM	4.88 +/- 0.63	4.85 –4.90	
50% Glycerol		4.56 +/-0.57	4.53 –4.60	<0.0001
	SubAB5 1 µg/ml	4.83 +/-0.67	4.81 –4.86	

B

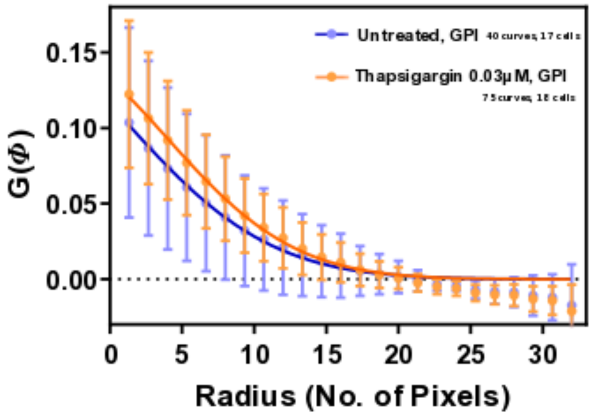

C

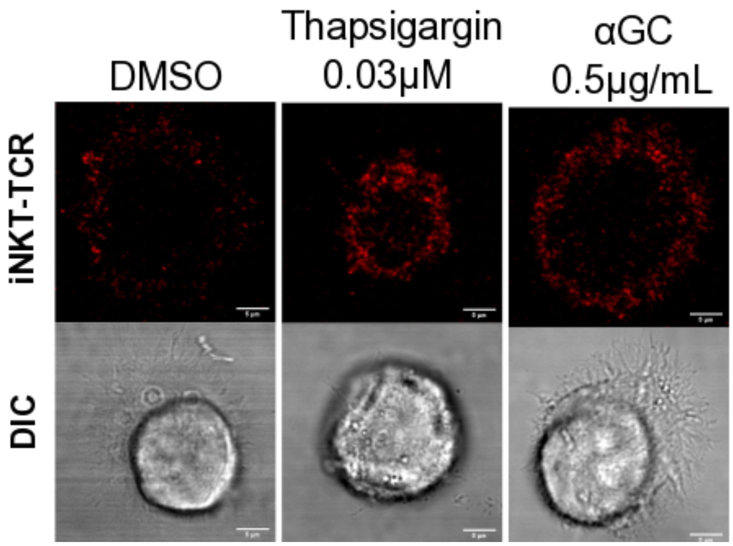

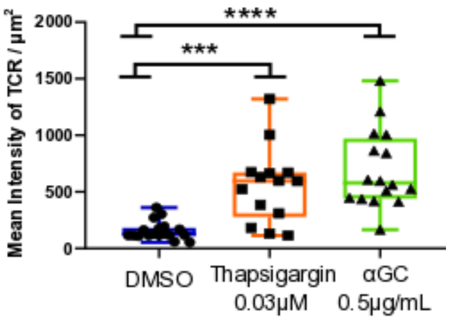

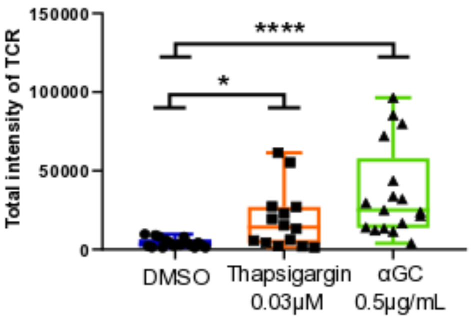

Figure S3: CD1d diffusion and iNKT-TCR staining in THP1 cells expressing WT and GPI-linked CD1d molecules. (A) Average diffusion times of CD1d molecules in THP1-CD1d cells treated with DMSO, thapsigargin or the SubAB5. The *p*-values showing statistical significance is shown for the ER-stress drugs and their corresponding control samples. **(B)** Quantification of heterogeneity in CD1d distribution on the surface of DMSO and thapsigargin-treated THP1-GPI-CD1d cells using the spatial autocorrelation function. **(C)** Enhanced binding of iNKT-TCR monomers to thapsigargin-treated THP1-CD1d cells compared to control treated cells. Top, confocal and DIC images; bottom, TCR mean intensity staining/μm^2 (left) and total intensity (right). *, ***, and **** represents $p<0.03$, $p<0.0005$ and $p<0.0001$, respectively, by one-way ANOVA with a Tukey's multiple comparisons post-test.

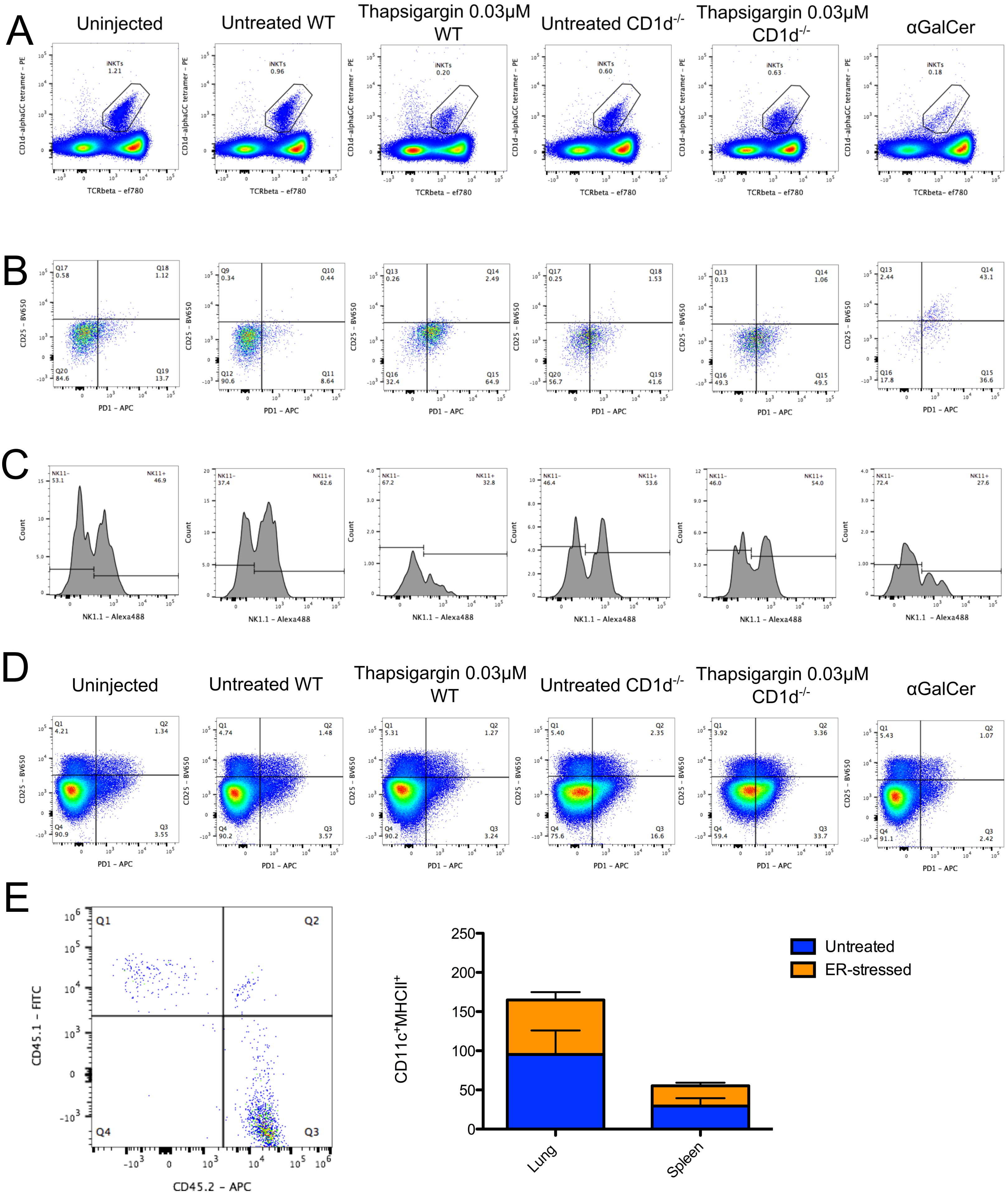
A
Uninjected
Untreated WT
Thapsigargin 0.03μM WT
Untreated CD1d-/-
Thapsigargin 0.03μM CD1d-/-
αGalCer
CD1d-alphaGC tetramer – PE
TCRbeta – ef780
iNKTs 1.21
iNKTs 0.96
iNKTs 0.20
iNKTs 0.60
iNKTs 0.63
iNKTs 0.18
B
CD25 – BV650
PD1 – APC
C
Count
NK1.1 – Alexa488
D
Uninjected
Untreated WT
Thapsigargin 0.03μM WT
Untreated CD1d-/-
Thapsigargin 0.03μM CD1d-/-
αGalCer
CD25 – BV650
PD1 – APC
E
CD45.1 – FITC
CD45.2 – APC
Q1
Q2
Q3
Q4
CD11c+MHCII+
Untreated
ER-stressed
Lung
Spleen

Figure S4: ER-stressed CD11c^{+} BMDCs trigger CD1d-dependent iNKT cell activation *in vivo.* **(A)** Dots plots indicating decreased frequency of CD1d-αGC tetramer, **(B)** PD1 and CD25 expression, and **(C)** NK1.1 expression on splenic iNKT cells. **(D)** PD1 and CD25 expression on non-iNKT CD4^{+} T cells in recipient mice. These are representative FACS plots of mice from one of three experiments described in Figure 6. **(E)** Gating used to distinguish CD45.1 and CD45.1/2 CD11c^{+} BMDCs injected into CD45.2 CD1d$^{-/-}$ recipient mice (left) and numbers of unstressed and ER-stressed CD11c^{+} BMDCs in the lungs versus the spleen of CD1d$^{-/-}$ recipient mice (right). The experiment was performed using N=6 recipient mice in a single independent experiment.

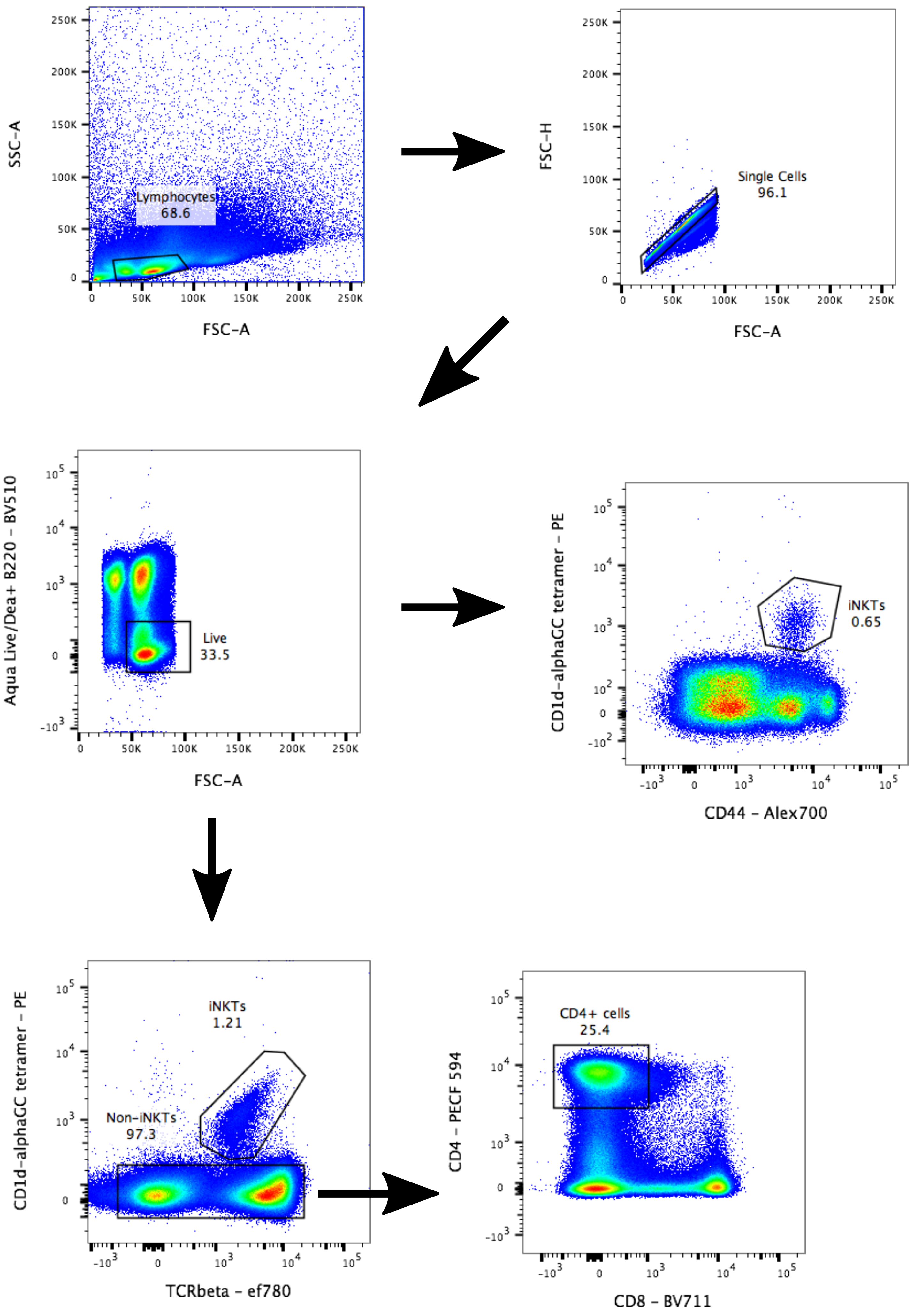

SSC-A
FSC-A
Lymphocytes
68.6
FSC-H
FSC-A
Single Cells
96.1
Aqua Live/Dea+ B220 - BV510
FSC-A
Live
33.5
CD1d-alphaGC tetramer - PE
CD44 - Alex700
iNKTs
0.65
CD1d-alphaGC tetramer - PE
TCRbeta - ef780
iNKTs
1.21
Non-iNKTs
97.3
CD4 - PECF 594
CD8 - BV711
CD4+ cells
25.4

Figure S5: Gating strategy for analyzing iNKT cells *in vivo*. Flow chart of the gating strategy used to analyze the iNKT cells in mice from Figure 8 based on an uninjected control mouse. Briefly, lymphocytes were gated based on forward and side scatter, then single cells on forward scatter area versus height, dead cells and B cells were excluded, and finally iNKT cells using the CD1d-αGC tetramer, from which expression levels of the activation markers CD25 and PD1 were determined. From the CD1d-αGC tetramer negative population, $CD4^+$ cells were analyzed for expression levels of the activation markers PD1 and CD25.

SI References

1. Salio M*, et al.* (2017) Activation of Human Mucosal-Associated Invariant T Cells Induces CD40L-Dependent Maturation of Monocyte-Derived and Primary Dendritic Cells. *Journal of immunology* 199(8):2631-2638.
2. Salio M*, et al.* (2013) Saposins modulate human invariant Natural Killer T cells self-reactivity and facilitate lipid exchange with CD1d molecules during antigen presentation. *Proceedings of the National Academy of Sciences of the United States of America* 110(49):E4753-4761.
3. Wild MK*, et al.* (1999) Dependence of T cell antigen recognition on the dimensions of an accessory receptor-ligand complex. *The Journal of experimental medicine* 190(1):31-41.
4. Liebisch G*, et al.* (2013) Shorthand notation for lipid structures derived from mass spectrometry. *Journal of lipid research* 54(6):1523-1530.
5. Schuhmann K*, et al.* (2012) Shotgun lipidomics on a LTQ Orbitrap mass spectrometer by successive switching between acquisition polarity modes. *J Mass Spectrom* 47(1):96-104.
6. Schuhmann K*, et al.* (2017) Monitoring Membrane Lipidome Turnover by Metabolic (15)N Labeling and Shotgun Ultra-High-Resolution Orbitrap Fourier Transform Mass Spectrometry. *Anal Chem* 89(23):12857-12865.
7. Schuhmann K*, et al.* (2017) Intensity-Independent Noise Filtering in FT MS and FT MS/MS Spectra for Shotgun Lipidomics. *Anal Chem* 89(13):7046-7052.
8. Herzog R*, et al.* (2011) A novel informatics concept for high-throughput shotgun lipidomics based on the molecular fragmentation query language. *Genome Biol* 12(1):R8.
9. Exley M*, et al.* (2000) CD1d structure and regulation on human thymocytes, peripheral blood T cells, B cells and monocytes. *Immunology* 100(1):37-47.
10. Waithe D*, et al.* (2018) Optimized processing and analysis of conventional confocal microscopy generated scanning FCS data. *Methods* 140-141:62-73.
11. Schneider F*, et al.* (2018) Statistical Analysis of Scanning Fluorescence Correlation Spectroscopy Data Differentiates Free from Hindered Diffusion. *ACS Nano* 12(8):8540-8546.
12. Fritzsche M, Erlenkamper C, Moeendarbary E, Charras G, & Kruse K (2016) Actin kinetics shapes cortical network structure and mechanics. *Sci Adv* 2(4):e1501337.
13. Fritzsche M & Charras G (2015) Dissecting protein reaction dynamics in living cells by fluorescence recovery after photobleaching. *Nat Protoc* 10(5):660-680.
